# Sugar import mediated by sugar transporters and cell wall invertases for seed development in *Camellia oleifera*

**DOI:** 10.1093/hr/uhae133

**Published:** 2024-05-10

**Authors:** Bingshuai Du, Yibo Cao, Jing Zhou, Yuqing Chen, Zhihua Ye, Yiming Huang, Xinyan Zhao, Xinhui Zou, Lingyun Zhang

**Affiliations:** State Key Laboratory of Efficient Production of Forest Resources, Key Laboratory of Forest Silviculture and Conservation of the Ministry of Education, The College of Forestry, Beijing Forestry University, No.35 Qinghua East Road, Haidian District, Beijing 100083, China; State Key Laboratory of Efficient Production of Forest Resources, Key Laboratory of Forest Silviculture and Conservation of the Ministry of Education, The College of Forestry, Beijing Forestry University, No.35 Qinghua East Road, Haidian District, Beijing 100083, China; State Key Laboratory of Efficient Production of Forest Resources, Key Laboratory of Forest Silviculture and Conservation of the Ministry of Education, The College of Forestry, Beijing Forestry University, No.35 Qinghua East Road, Haidian District, Beijing 100083, China; State Key Laboratory of Efficient Production of Forest Resources, Key Laboratory of Forest Silviculture and Conservation of the Ministry of Education, The College of Forestry, Beijing Forestry University, No.35 Qinghua East Road, Haidian District, Beijing 100083, China; State Key Laboratory of Efficient Production of Forest Resources, Key Laboratory of Forest Silviculture and Conservation of the Ministry of Education, The College of Forestry, Beijing Forestry University, No.35 Qinghua East Road, Haidian District, Beijing 100083, China; State Key Laboratory of Efficient Production of Forest Resources, Key Laboratory of Forest Silviculture and Conservation of the Ministry of Education, The College of Forestry, Beijing Forestry University, No.35 Qinghua East Road, Haidian District, Beijing 100083, China; State Key Laboratory of Efficient Production of Forest Resources, Key Laboratory of Forest Silviculture and Conservation of the Ministry of Education, The College of Forestry, Beijing Forestry University, No.35 Qinghua East Road, Haidian District, Beijing 100083, China; State Key Laboratory of Efficient Production of Forest Resources, Key Laboratory of Forest Silviculture and Conservation of the Ministry of Education, The College of Forestry, Beijing Forestry University, No.35 Qinghua East Road, Haidian District, Beijing 100083, China; State Key Laboratory of Efficient Production of Forest Resources, Key Laboratory of Forest Silviculture and Conservation of the Ministry of Education, The College of Forestry, Beijing Forestry University, No.35 Qinghua East Road, Haidian District, Beijing 100083, China

## Abstract

Seed development and yield depend on the transport and supply of sugar. However, an insufficient supply of nutrients from maternal tissues to embryos results in seed abortion and yield reduction in *Camellia oleifera*. In this study, we systematically examined the route and regulatory mechanisms of sugar import into developing *C. oleifera* seeds using a combination of histological observations, transcriptome profiling, and functional analysis. Labelling with the tracer carboxyfluorescein revealed a symplasmic route in the integument and an apoplasmic route for postphloem transport at the maternal–filial interface. Enzymatic activity and histological observation showed that at early stages [180–220 days after pollination (DAP)] of embryo differentiation, the high hexose/sucrose ratio was primarily mediated by acid invertases, and the micropylar endosperm/suspensor provides a channel for sugar import. Through *Camellia* genomic profiling, we identified three plasma membrane-localized proteins including CoSWEET1b, CoSWEET15, and CoSUT2 and one tonoplast-localized protein CoSWEET2a in seeds and verified their ability to transport various sugars via transformation in yeast mutants and calli. *In situ* hybridization and profiling of glycometabolism-related enzymes further demonstrated that *CoSWEET15* functions as a micropylar endosperm-specific gene, together with the cell wall acid invertase CoCWIN9, to support early embryo development, while *CoSWEET1b*, *CoSWEET2a*, and *CoSUT2* function at transfer cells and chalazal nucellus coupled with CoCWIN9 and CoCWIN11 responsible for sugar entry in bulk into the filial tissue. Collectively, our findings provide the first comprehensive evidence of the molecular regulation of sugar import into and within *C. oleifera* seeds and provide a new target for manipulating seed development.

## Introduction

Seed growth and development are important steps in the completion of the plant life cycle and are key to realizing the potential for crop yields, which depend on the efficient supply and utilization of sugar for developing seeds. As a female reproductive tissue, the seed that develops from an ovule in angiosperms after double fertilization is a major storage organ in plants, determining crop productivity and quality [1, 2]. Based on the ultimate morphology of seed development, angiosperm plants are usually divided into two types: monocotyledons, with a single cotyledon, and dicotyledons, with two cotyledons in most dicot species [3]. In dicotyledonous plants, the endosperm as an intermediate tissue completes the transfer of nutrients to the embryo, especially sugars as a major source of energy and carbon skeletons for cell wall and starch biosynthesis [4, 5], while the nutrient supply for the endosperm relies on the transportation of nutrients from maternal tissue to filial seeds [6].

Sucrose, the dominant transport form of photoassimilates in most plants, is translocated from source leaves via the phloem by pressure-driven bulk flow and is subsequently unloaded into a wide range of heterotrophic sink organs, such as fruits or seeds, to support plant development [7–9]. The routes by which sucrose is unloaded from the phloem vary with plant tissue and developmental stage, including symplasmic transport, the apoplasmic pathway, or a combination of the two [10]. In seeds, sugar transport consecutively traverses three genomically distinct domains: maternal pericarp/seed coat, filial endosperm, and filial embryo. Due to the absence of symplasmic connections between maternal and filial tissues, sucrose is usually translocated via an apoplasmic step for phloem unloading and postphloem transport into the seed [8, 11]. During this process, various sugar transporters, such as sugar transporter proteins (STPs), sucrose transporters (SUTs or SUCs), and uniporters (SWEETs, the sugar will eventually be exported transporters), play a crucial role in sugar transport [12–14].

SUTs belong to the major facilitator superfamily and function as sucrose/proton symporters depending on membrane energization [15]. In recent years, many studies have reported that SUTs are involved in the regulation of fruit saccharinity and crop in many plant species, such as *Solanum lycopersicum* [16, 17], *Pyrus lindleyi* [18], *Malus pumila* [19], *Oryza sativa* [20], *Glycine max* [21], and *Zea mays* [22]. Unlike SUTs, SWEETs are bidirectional sugar transporters that facilitate both the efflux and influx of monosaccharides and/or disaccharides, depending on the sugar concentration on either side of the membrane [23, 24]. SWEET proteins are generally divided into four clades: clades I and II, whose members are mainly responsible for hexose transport; clade III, whose members preferentially transport sucrose; and clade IV, whose members act on the flux of fructose across the tonoplast [25, 26]. Recently, SWEET proteins have been reported to play critical roles in biomass production and seed filling in plants [7, 27]. Since most SWEETs are hexose transporters, they are usually required to work cooperatively with cell wall invertases (CWINs) to modulate sugar allocation and signalling in plants [28]. Invertases comprise CWINs, cytoplasmic invertases (CINs) and vacuolar invertases (VINs), all of which can split sucrose into glucose and fructose at their corresponding cell locations [29]. Especially, *CWIN* transcript signals have been detected and function coupled with sugar transporters during seed development across a wide range of species, including *Z. mays* [30], *Arabidopsis* [31], *Gossypium hirsutum* [29], and *O. sativa* [32]. However, the regulatory mechanisms of sugar translocation in the seed have only been revealed in model plant and a very limited number of crop species, such as cotton, maize, and faba bean seeds [8, 29, 33]. The more complex and specific mechanisms of sugar import into the seed are in urgent need of elucidation in plant species, which is expected to provide new insights into seed abortion in different types.

*Camellia oleifera* Abel. is considered as one of the four major woody edible oil plants worldwide and is widely distributed in southern China [34, 35]. *C. oleifera* seeds are primarily composed of a mature embryo that produces high-quality edible oil with as much as 90% unsaturated fatty acid and no less than 80% oleic acid [35, 36]. However, severe seed abortion and dysplasia limit seed production, and up to 70% of the current area planted with oil tea plants is low-seed-yield forests that struggle to meet the growing human demand for tea oil [37]. As a typical dicotyledon plant, the development and accumulation of storage products in the embryo depend on the efficient unloading of assimilates from the terminal phloem in the maternal tissue and the supply of nutrients from the endosperm in *C. oleifera*. Although there have been several reports of the identification of genes potentially involved in sugar transport in *C. oleifera* fruits based on transcriptome analysis [38, 39], the knowledge of the regulatory mechanism for sugar import into the seed is still very limited.

In this study, we attempted to gain a comprehensive understanding of phloem unloading and postphloem sugar transport coupled with sugar metabolism in developing *C. oleifera* seeds. By using microscopy observation and fluorescent labelling, we captured the morphogenesis process of the embryo and endosperm, revealing the sugar transport route and storage during seed morphogenesis, and constructed a nexus of nutritional dynamics and seed development. Based on the genomics of wild oil *Camellia* and *C. lanceoleosa* [35, 40], we performed an in-depth data analysis of spatio-temporal expression profiles of sugar transporters and metabolizing enzymes that are potentially involved in the import and translocation of assimilates in seeds and identified key candidates such as CoSWEET1b, CoSWEET2a, CoSWEET15, and CoSUT2, as well as the invertases CoCWIN9 and CoCWIN11. *In situ* hybridization and functional analysis further revealed the action sites and transport characteristics of these proteins in seed development. Collectively, we first establish a hypothetical model of the spatiotemporal control of sugar transport and partitioning regulated cooperatively by sugar transporters and sucrose-metabolizing enzymes in *C. oleifera* seeds and deepen our understanding of nutritional regulation during seed development in dicotyledonous plants.

## Results

### Microscopic observation of the embryo and endosperm during seed development

**Figure 1:**
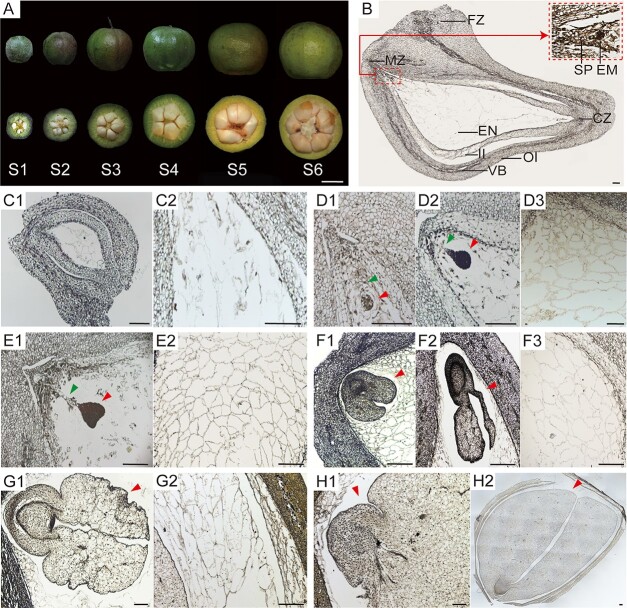
Morphological changes in *C. oleifera* seeds at different developmental stages. (A) Dynamic changes in *C. oleifera* fruit and seed development under a stereomicroscope. (B) Complete microstructure of a longitudinal section of *C. oleifera* seeds, showing the anatropous ovule. The red box represents a magnified view of the selected area, showing the embryo and suspensor. (C–H) Morphological changes in the embryo and endosperm during seed morphogenesis. (C1, C2) were sampled at stage S1, (D1–D3) at stage S2, (E1, E2) at stage S3, (F1–F3) at stage S4, (G1–G2) at stage S5, and (H1, H2) at stage S6. The red arrows point to the embryos and the green arrows point to the suspensors. S1–S6 represent stage 1 (S1, 180 DAP), stage 2 (S2, 200 DAP), stage 3 (S3, 220 DAP), stage 4 (S4, 240 DAP), stage 5 (S5, 260 DAP), and stage 6 (S6, 280 DAP), respectively. FZ, funicle zone; MZ, micropyle zone; SP, suspensor; EM, embryo; EN, endosperm; CZ, chalaza zone; II, inner integument; OI, outer integument; VB, vascular bundle. The scale bar in A is 1 cm, and the scale bar in B-H2 is 100 μm.

To gain a basic understanding of seed development dynamics in *C. oleifera*, six stages (S1–S6) were established during fruit and seed development based on the developmental characteristics of the embryo and the endosperm. The rapid enlargement of the fruit was accompanied by a marked increase in fresh weight and transverse and longitudinal diameter (Fig. 1A and Supplementary Fig. S1). *C. oleifera* seeds are typically anatropous ovules and can be divided into micropyle, funicle, and chalazal zones, with vascular bundles distributed in the outer integument (Fig. 1B). At stage 1 (S1, 180 DAP), the endosperm nuclei were observed to be distributed within the embryo sac, at which point the embryo body was too small to discern (Fig. 1C1 and C2). At stage 2 (S2, 200 DAP), the embryo was in the spherical stage, and the endosperm nuclei underwent cellularization from the micropyle end to the chalazal end, while the suspensor was fully developed, probably serving as a channel for the transport of nutrients to the embryo bodies (Fig. 1D1 and D3). At stage 3 (S3, 220 DAP), the embryo was heart-shaped, and the endosperm cells were widely distributed throughout the embryo sac (Fig. 1E1 and E2). At stage 4 (S4, 240 DAP), the embryo continued to develop into a torpedo or cotyledonary shape, and the endosperm cells fully developed, at which point the suspensor disappeared (Fig. 1F1 and F2). At stage 5 (S5, 260 DAP), the cotyledon embryo continued to expand, and the endosperm was gradually absorbed by the embryo for nutrients and space (Fig. 1G1 and G2). At stage 6 (S6, 280 DAP), the endosperm was almost entirely absorbed, and the fully expanded cotyledon occupied the entire embryo sac (Fig. 1H1 and H2). Overall, it takes ~3 months from embryonic development to the end of morphological differentiation in *C. oleifera* seeds.

### Dynamic changes and accumulation of assimilates in the compartments of *C. oleifera* seeds

**Figure 2:**
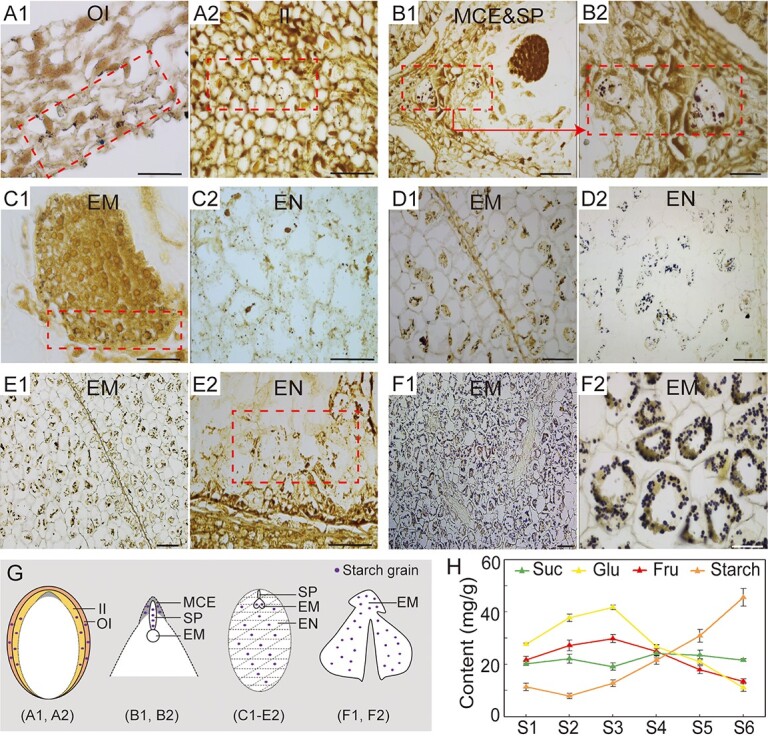
The accumulation of starch granules and sugar content in developing *C. oleifera* seeds. (A1, A2) Starch grains were observed in the seeds of stage 1 (180 DAP) and mainly accumulated in the seed coat. (B1, B2) Starch grains in the seeds of stage 2 (200 DAP) and mainly accumulated in the micropylar endosperm cells and suspensor, and B2 is an enlarged view of B1. (C1, C2) Starch grains in the seeds of stage 3 (220 DAP) mainly accumulated in the endosperm and outside of the embryo. (D1, D2) Starch grains were present in stage 4 (240 DAP) seeds and exhibited obvious increases in both the embryo and endosperm. (E1, E2) Starch grains in seeds of stage 5 (260 DAP) seeds accumulated in the enlarged cotyledon embryo, with a few remaining in the apoptotic endosperm cells. (F1, F2) Starch grains were observed in stage 6 (280 DAP) seeds and continued to aggregate in the enlarged cotyledon embryos. F2 is an enlarged view of F1. (G) A mode of dynamic changes in starch storage during seed morphogenesis, showing the starch locations for A1-F2. (H) The contents of sugar and starch at different stages in *C. oleifera* seeds. OI, outer integument; II, inner integument; MCE, micropylar cellularizing endosperm; SP, suspensor; EM, embryo; EN, endosperm; Suc, sucrose; Glu, glucose; Fru, fructose. The scale bar is 50 μm.

In a previous study, photoassimilates were demonstrated to be transported in the form of sucrose from source leaves to sinks in *C. oleifera* [41], and excess carbon was stored as starch in fruits to support seed development [42]. To reveal the nutritional dynamics and specific sites of sugar accumulation in the compartments of seeds during fruit development, we stained the seed sections and observed the distribution of starch granules. Only a small amount of starch was transiently stored in the outer and inner integuments at stage 1 (180 DAP) (Fig. 2A1 and A2). At stage 2 (200 DAP), starch was observed in the micropylar endosperm and suspensor (Fig. 2B1 and B2). At stage 3 (220 DAP), a few starch grains were present in the embryo (Fig. 2C1), and more starch granules accumulated in the endosperm during the cellularization of the endosperm (Fig. 2C2). With seed development (S4, 240 DAP), large amounts of starch granules were accreted both in the developing embryo and in the endosperm (Fig. 2D1 and D2). When the seeds had completed morphological differentiation (S5, 260 DAP), the starch granules were primarily distributed within the cotyledon embryo, at which point only a small amount of starch was retained in the endosperm cells, which were undergoing apoptosis (Fig. 2E1 and E2). At stage 6 (280 DAP), starch continued to accumulate rapidly with cotyledon embryo expansion (Fig. 2F1 and F2). Based on these results, we sketched the dynamic pattern of assimilate transport and accumulation during *C. oleifera* seed morphogenesis, showing (i) the transfer from the seed coat to the micropylar endosperm and suspensor, (ii) the transfer to endosperm cells, and (iii) the final translocation to the enlarged cotyledon from the apoptotic endosperm cells and deposit (Fig. 2G).

Determination of starch content further verified that the starch content decreased in early stages and reached a minimum at S2 with only ~7.86 mg/g, and then rapidly increased, reaching 45.54 mg/g at S6 (Fig. 2H), which was consistent with the staining results of the seed sections. The soluble sugar content decreased when starch rapidly accumulated, during which the fructose content declined the most obviously, followed by that of glucose. Notably, the hexose-to-sucrose ratio first increased rapidly from 2.43 at S1 to ~3.75 at S3 and then decreased to only 1.13 at S6 (Fig. S2). These results suggest that sucrose decomposition is the dominant event in the early stage of embryo development and that the storage products begin to accumulate upon completion of cotyledonary embryo differentiation.

### CF is confined to the vascular tissue of the seed coat and absent in the filial tissues

**Figure 3:**
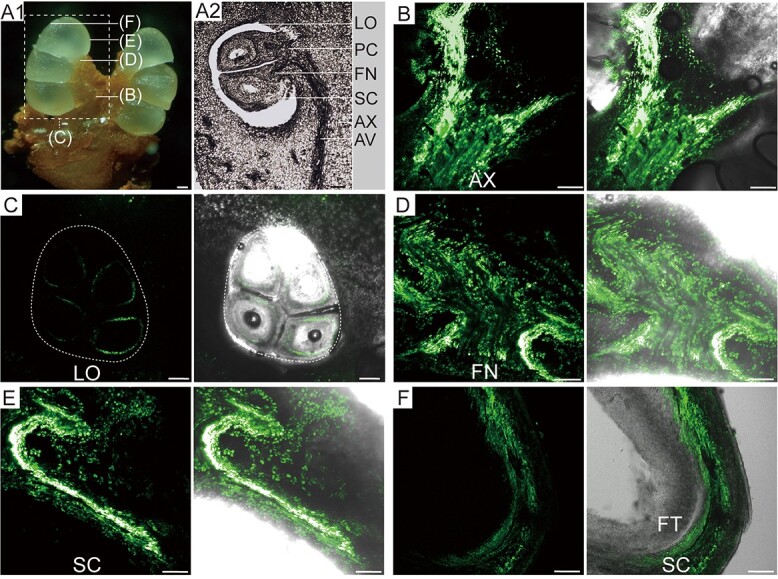
CF-traced sugar transport pathway in *C. oleifera* seeds. (A1) Morphological observation of *C. oleifera* fruit, showing the sampling locations for B–F. (A2) Longitudinal sections of the fruits, showing the distribution of vascular bundles connecting seeds. (B) Confocal imaging of CF transport from the axile to the placenta. CF fluorescence was widely distributed in the vascular tissue and adjacent cells. (C) Confocal imaging of CF transport in the locule. The fluorescence of CF was confined to the outermost layer of seeds. (D) Confocal imaging of CF transport in the funicle zone of seeds. CF fluorescence was widely distributed in vascular tissue and adjacent cells. (E) Confocal imaging of CF transport from the funicle to the seed coat. CF can be transferred unreservedly into the seed coat. (F) Confocal imaging of CF transport in seeds showing that CF was confined to the vascular tissue of the seed coat but absent in the filial tissues. Seeds and fruits at 20 DAP were sampled to observe the microstructure and CF transport in A–C; The seeds and fruits at 220 DAP were used for the observation of CF transport in D–F. LO, locule; FN, funicle; PC, placenta; SC, seed coat; AX, axile; AV, axile vascular bundle; FT, filial tissues. The white arrows represent the transport direction of assimilates. The scale bar is 100 μm.

The efficient unloading of assimilates from the terminal phloem in the maternal to filial tissue determines seed development and crop productivity. To explore phloem unloading and the transfer of assimilates among compartments of *C. oleifera* seeds, a fluorescent tracing experiment was conducted to validate the route of sugar unloading into and within seeds. The fluorescent tracer carboxyfluorescein (CF) can move only via plasmodesmata in the cell, and its fluorescence reveals symplasmically connected compartments [43]. Here, the distribution of vascular bundles in *C. oleifera* fruits was revealed by observation of longitudinal or transverse sections (Fig. S3A, B and Fig. 3A1, A2). Briefly, ~5 carpels were observed to approach and attach to the central column of fruit, forming axile placentation (Fig. S3A and B). The longitudinal section of the fruit showed the placental bundle, to which the vascular bundle of the axile attaches at one end and the funicle at the other end (Fig. 3A2). Confocal laser scanning microscopy (CLSM) images of CF movement sampled from the region between the axile and the funicle at the very early stage of fruit development (20 DAP) showed that CF was widely distributed in the vascular tissue and adjacent cells of axile and placenta (Fig. 3B). However, in the ventricles, the CF was strictly confined to the outermost layer of the seeds (Fig. 3C). With fruit development, CF was still distributed in the vascular tissue and adjacent cells of the funicle at 220 DAP (Fig. 3D) and could be transferred unreservedly from the funicle to the seed coat via vascular bundles (Fig. 3E) but confined to the seed coat and absent in the filial tissues (Fig. 3F). These results demonstrate that symplasmic isolation occurs between the seed coat and filial tissue throughout entire seed development and that apoplasmic pathway is employed for assimilate transport into the seed of *C. oleifera*.

### Screening and identification of sugar transporters involved in assimilate import in seeds

**Figure 4:**
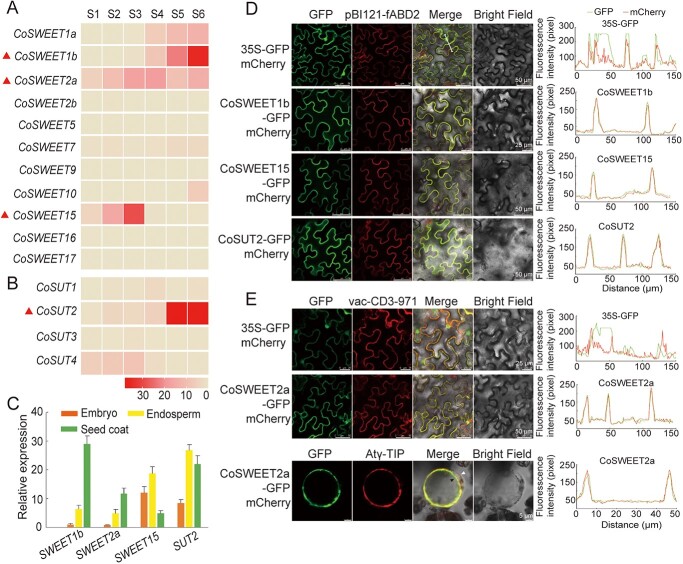
The expression profile and subcellular location of *CoSWEET1b*, *2a*, *15*, and *CoSUT2*. (A, B) The expression profiles of the *CoSWEET* and *CoSUT* transporters during seed morphogenesis. *CoSWEET1b*, *CoSWEET2a*, *CoSWEET15*, and *CoSUT2* had higher expression levels than did the other genes. The *CoGAPDH* (glyceraldehyde-3-phosphate dehydrogenase) gene was used as an internal reference. (**C**) Tissue-specific expression of *CoSWEET1b*/*2a*/*15* and *CoSUT2* in the embryo, endosperm and seed coat of *C. oleifera* seeds. Different tissues were collected at 240 DAP. The data are shown as the means ± SDs of three independent biological replicates. (D) The subcellular location of CoSWEET1b, CoSWEET15, and CoSUT2. pBI121-fABD2 is a plasma membrane marker. Microscopy analysis showed that CoSWEET1b, CoSWEET15, and CoSUT2 are located in the plasma membrane. The white line indicates the profile type and location of data acquisition in the fluorescence quantitative map on the right. (E) Subcellular localization of CoSWEET2a-GFP fusion proteins in the epidermis cells and protoplasts of tobacco. The constructs carrying vac-CD3-971 and Atγ-TIP were used as tonoplast-localized marker, respectively. CoSWEET2a is located in the tonoplast. The white arrow points to the plasma membrane, and the black arrow points to the tonoplast.

For the apoplasmic route, the exit of sucrose into the apoplasmic space and subsequently into the cell is facilitated by various sugar transporters [21]. To identify the potential genes responsible for sugar import into *C. oleifera* seeds, we screened the *C. oleifera* genome database (No. PRJNA732216) [35] and identified 11 CoSWEETs, 4 CoSUTs, and 8 CoSTPs via homology-based BLAST. These genes were classified and named according to the topology of the phylogenetic tree and the categories of SWEETs, SUTs, and STPs in *Arabidopsis* (Fig. S4). qRT-PCR assays revealed that *CoSWEET15* was highly expressed in the early stages (S1–S3). *CoSWEET1b* and *CoSUT2* were specifically expressed in the late stages (S4–S6), and *CoSWEET2a* was expressed in almost all stages (Fig. 4A and B). However, the transcript levels of all of the evaluated *CoSTP* genes remained consistently low from S1 to S6 (Fig. S5). Phylogenetic analysis revealed that CoSWEET1b and CoSWEET2a belong to Clade I, while CoSWEET15 belongs to Clade III (Fig. S4). Tissue-specific expression showed that *CoSWEET1b*, *CoSWEET15* and *CoSUT2* had markedly greater mRNA levels in seeds than in other tissues, whereas *CoSWEET2a* was highly expressed in multiple tissues including flowers, seeds, roots, and mature leaves (Fig. S6). We further determined the expression levels of these genes in different seed compartments and found that *CoSWEET1b* and *CoSWEET2a* were preferentially expressed in the seed coat, *CoSWEET15* mRNA levels were higher in the embryo and endosperm, and *CoSUT2* was relatively highly expressed in the endosperm and seed coat (Fig. 4C). These results suggest that the four sugar transporter genes probably function at different sites in seeds and were therefore selected as key candidates for further analysis.

To confirm the intracellular localization of *CoSWEET1b*, *2a*, *15*, and *CoSUT2*, the coding sequence (CDS) of the four genes were combined with GFP under the control of the 35S promoter. The co-expressed pCAM35S::CoSWEET1b-GFP, pCAM35S::CoSWEET2a-GFP, pCAM35S::CoSWEET15-GFP or pCAM35S::CoSUT2-GFP were then introduced into the epidermal cells of *Nicotiana benthamiana* leaves along with an mCherry-labelled plasma membrane marker (pBI121-fABD2) or tonoplast marker protein (vac-CD3-971). The red fluorescence of pBI121-mCherry-fABD2 overlapped with the green fluorescence of CoSWEET1b, CoSWEET15, and CoSUT2-GFP, indicating that CoSWEET1b, CoSWEET15, and CoSUT2 were localized in the plasma membrane (Fig. 4D). The overlap of the red fluorescence of vac-CD3-971 with the green fluorescence of CoSWEET2a-GFP in tobacco leaves suggested the localization of CoSWEET2a in the tonoplast (Fig. 4E). The co-localization of CoSWEET2a with the tonoplast marker Atγ-TIP in tobacco protoplasts further demonstrated that CoSWEET2a is a tonoplast-localized transporter.

### Transport properties and response to sugar for CoSWEET1b, 2a, 15, and CoSUT2

**Figure 5:**
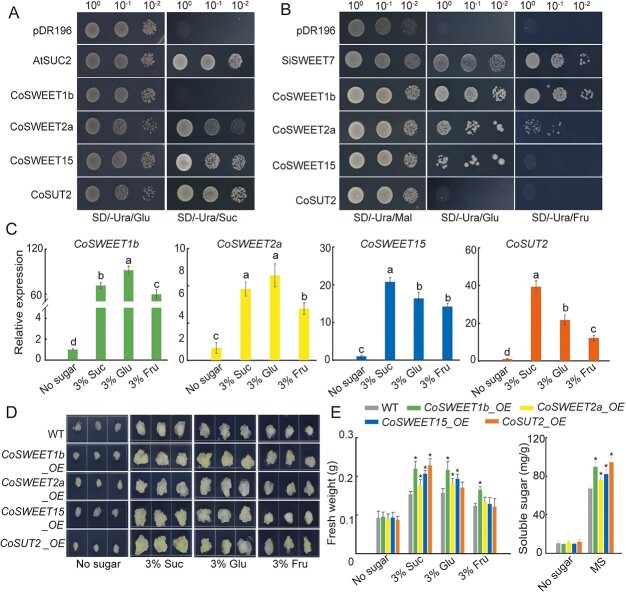
Functional determination of CoSWEET1b, 2a, 15, and CoSUT2 in yeast mutant, calli and *C. oleifera* seeds. (A) Complementation assays of sucrose transport deficiency by CoSWEET1b, 2a, 15, and CoSUT2 in the yeast mutant strain SUSY7/ura3. Yeast mutant strains transformed with AtSUC2 were used as a positive control, and the pDR196 empty vector was used as a negative control. (B) Complementation assays of hexoses transport deficiency by CoSWEET1b, 2a, 15, and CoSUT2 in yeast mutant strain EBY.VW4000. Yeast mutant strains transformed with SlSWEET7a were used as positive controls, and the strains transformed with pDR196 empty vector were used as negative controls. (C) Relative expression levels of the four genes *in vitro* culture of *C. oleifera* seeds on medium containing 3% sucrose, 3% glucose, and 3% fructose, respectively. Transcript levels were determined by performing qRT-PCR relative to *CoEF1α*. (D) Phenotypes observation of WT and transgenic apple calli cultured *in vitro* on media supplemented with 3% sucrose, 3% glucose, and 3% fructose, respectively. (E) The fresh weight and soluble sugar content of WT and transgenic calli. Significant differences were determined by one-way ANOVA followed by Duncan’s multiple comparisons test (*P* < 0.05) in (C) and (E). Suc, sucrose; Glu, glucose; Fru, fructose; Mal, maltose.

To further characterize the transport properties of CoSWEET1b, 2a, 15, and CoSUT2, we determined the ability of the four sugar transporters to take up sucrose and hexose via the heterologous expression of their respective cDNAs in the mutant yeast cells (*Saccharomyces cerevisiae*) strain SUSY7/ura3, which is deficient in sucrose uptake [44], and the strain EBY.VW4000, which is deficient in hexose uptake [45]. On the medium supplemented with sucrose, the expression of CoSWEET2a, CoSWEET15, CoSUT2, and AtSUC2 (used as a positive control) restored SUSY7/ura3 growth, but CoSWEET1b did not (Fig. 5A). On the medium supplemented with glucose as a carbon source, the mutant EBY.VW4000 harbouring CoSWEET1b, 2a, and 15 grew normally (Fig. 5B). Only yeast cells harbouring CoSWEET1b and 2a could grow normally on medium supplemented with fructose as a carbon source (Fig. 5B). These results indicate that CoSWEET1b can transport glucose and fructose; CoSWEET2a can transport sucrose, glucose and fructose; CoSWEET15 can transport sucrose and glucose; and CoSUT2 can only transport sucrose.

Previous studies have shown that plant vacuole-localized transporters are likely to be altered in their expression sites when heterogeneously expressed in yeast, such as AtSUT4 [46], MdERDL6 [47], and VvVPT1 [48]. In order to confirm whether the yeast system is effective for the transport properties of CoSWEET2a, we first detected its localization in yeast by expressing the CoSWEET2a-GFP-pDR196 fusion vector in the yeast mutant EBY.VW4000. The results showed that CoSWEET2a could be expressed in yeast system and was not confined to the tonoplast membrane (Fig. S7). Furthermore, a mimic uptake of glucose and sucrose in yeasts via 2-[N-(7-nitrobenz-2-oxa-1,3-diazol-4-yl) amino]-2-deoxy-d-Glc (2-NBDG) and esculin showed that the positive strains containing CoSWEET2a were able to take up 2-NBDG and esculin and appeared with fluorescence (Fig. S8). These results suggest that the transport properties of CoSWEET2a in yeast are valid.

To determine the effects of different kinds of sugar and potential sugar signals on the transcription levels of the four genes, *C. oleifera* seeds were cultured *in vitro* on different medium containing 3% sucrose, 3% glucose, and 3% fructose. In comparison with those in the control group, the transcript abundances of the four genes in the treatment group exhibited obvious increases under the different kinds of sugar (Fig. 5C). In particular, the expression levels of *CoSWEET1b* and *CoSWEET2a* were greater under glucose treatment than under the other sugar treatments. In medium containing sucrose, the transcript levels of *CoSWEET15* and *CoSUT2* substantially increased.

To further explore the functional characteristics of the four sugar transporters, we transferred these genes into calli to obtain the stable transformed calli (Fig. S9A and B). qRT-PCR assays revealed that the *CoSWEET1b*, *2a*, *15*, and *CoSUT2*-overexpression (OE) lines produced significantly more transcripts than the wild-type (WT) calli (Fig. S9C). Phenotypic assay showed that no obvious differences were observed between the WT and transgenic calli on medium without sugars. However, when cultured on the medium supplemented with 3% sucrose, the calli overexpressed with these genes showed a significant increase in fresh weight compared to WT, most notably in the *CoSUT2-OE* calli (0.23 g), followed by *CoSWEET1b-OE* calli (0.22 g) (Fig. 5D). Under 3% glucose culture conditions, the fresh weight of the *CoSWEET1b-OE*, *CoSWEET2a-OE*, and *CoSWEET15-OE* calli were 37.6%, 15.8%, and 23.1% higher than that of WT, respectively, while no significant difference was observed between *CoSUT2-OE* calli and WT. Under 3% fructose culture conditions, only *CoSWEET1b-OE* showed a significant increase in fresh weight compared to WT. Moreover, the contents of soluble sugar were greatly increased in all transgenic calli compared to WT (Fig. 5E). These results suggest that these sugar transporters can respond positively to different kinds of sugar treatments and sugar signals, thus contributing to sugar transport and cell growth.

### CoSWEET1b, 2a, 15, and CoSUT2 function in the chalazal nucellus, micropylar cellularizing endosperm and transfer cells of *C. oleifera* seeds

**Figure 6:**
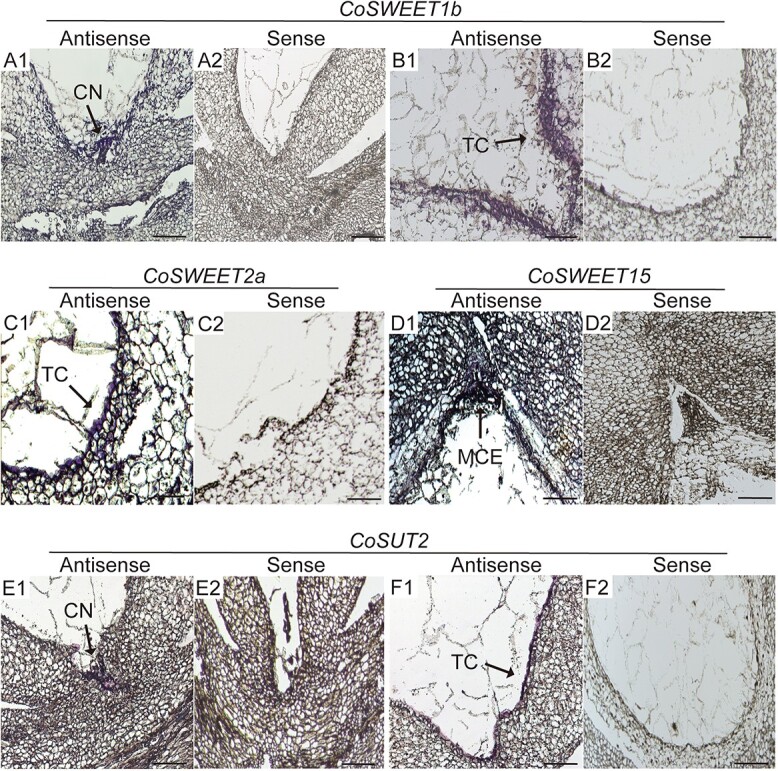
*In situ* hybridization of *CoSWEET1b*, *2a*, *15*, and *CoSUT2* in *C. oleifera* seeds. The seeds were longitudinally sectioned. (A1–B2) *In situ* hybridization with *CoSWEET1b*-antisense probe, which showed strong expression in the chalazal nucellus (A1) and in the transfer cells (B1). (A2, B2) A negative control with a *CoSWEET1b*-sense probe produced a very low or no background signal. (C1) *In situ* hybridization with a *CoSWEET2a*-antisense probe, which showed strong expression in transfer cells. (C2) A negative control with a *CoSWEET2a*-sense probe showed low or no background signal. (D1) *In situ* hybridization with the antisense probe of *CoSWEET1*5, showing strong signal in micropylar cellularizing endosperm. (D2) Negative control with a *CoSWEET15*-sense probe showed very low or no background signal. (E1–F2) *In situ* hybridization with antisense probe of *CoSUT2*, showing strong expression in the chalazal nucellus (E1) and in the transfer cells (F1), whereas the negative control with the *CoSUT2*-sense probe showed very low or no background signal (E2, F2). CN, chalazal nucellus; TC, transfer cells; MCE, micropylar cellularizing endosperm. The scale bar is 50 μm.

Since *CoSWEET1b, 2a, 15, and CoSUT2* had the highest expression levels during seed development and because they transport different kinds of sugars, the unloading sites of assimilates regulated by sugar transporters were determined by localizing transcripts of the four genes in *C. oleifera* seeds. Seeds at 200–240 DAP with endosperm cellularization in progress or with a relatively complete morphological structure were selected for *in situ* hybridization assays. As shown in Fig. 6, longitudinal sections of seeds were hybridized to specific antisense probes for *CoSWEET1b*, *2a*, *15*, and *CoSUT2*, while the sense strands corresponding to those genes were used as negative controls. The mRNA of *CoSWEET1b* and *CoSUT2* were mainly localized in transfer cells (at the innermost seed coat cell layer) and in the chalazal nucellus (Fig. 6A1, B1, E1, and F1), whereas *CoSWEET2a* transcript was only observed in transfer cells (Fig. 6C1). For *CoSWEET15*, the positive signals were observed in the micropylar cellularizing endosperm (Fig. 6D1) of seeds at 200 DAP, but they disappeared in the seeds at 240 DAP (Fig. S10). Notably, no signals were found for any of these genes in the sections treated with the sense probes (Fig. 6A2, B2, C2, D2, E2, and F2), suggesting the validity and reliability of the results.

### Compartment- and stage-specific expressions of glycometabolic enzymes and CWIN-coupled hexose uptake in seeds

**Figure 7:**
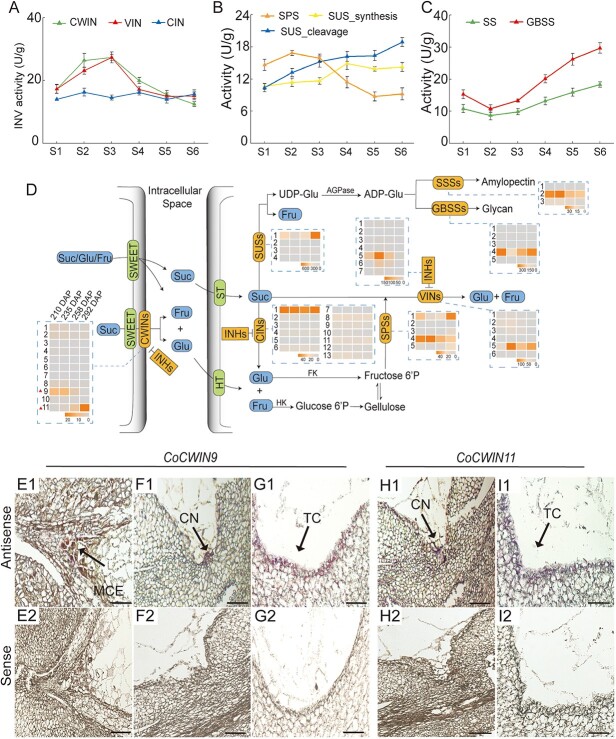
The activity of glycometabolism-related enzymes and spatial–temporal expression of the key genes in *C. oleifera* seeds. (A) Determination of invertase activity, including cell wall invertase (CWIN), vacuole invertase (VIN), CIN. (B) Activity assays of sucrose synthase (SUS), SPS. (C) Activity assays of starch synthase (SSS) and GBSS. (D) The screening of key genes in response to glycometabolism during *C. oleifera* seeds morphogenesis, such as *CWINs*, *VINs*, *CINs*, *INHs*, *SUSs*, *SPSs*, *SSSs* and *GBSSs*, which were shown in the form of metabolic map. Suc, sucrose; Glu, glucose; Fru, fructose; ST, sucrose transporter; HT, hexose transporter. (E1, F1, G1) *In situ* hybridization with a *CoCWIN9*-antisense probe. The longitudinal sections of *C. oleifera* seeds were hybridized to the specific antisense probes for *CoCWIN9*, showing strong expression in the chalazal nucellus, micropylar cellularizing endosperm and the transfer cells. (H1, I1) *In situ* hybridization with a *CoCWIN11*-antisense probe, which showed strong expression in the chalazal nucellus and in the transfer cells. (E2, F2, G2, H2, I2) A negative control with a *CoCWIN9* and *11*-sense probe, which showed very low or no background signal. CN, chalazal nucellus; TC, transfer cells; MCE, micropylar cellularizing endosperm. The scale bar is 50 μm.

Sugar transport and accumulation are usually coordinated with sucrose metabolism during seed development. Since high levels of hexose can promote cell division and inhibit the accumulation of storage material during early seed development, we first focused to examine the activities of sucrose-degrading enzymes, namely sucrose synthase (SUS) and invertases (INV). Here, we found that CWIN and VIN had greater activity in the early stages of seed development, peaking at S3, and then decreasing, while that of CIN was consistently low from S1 to S6 (Fig. 7A). In comparison, SUS activity in the cleavage direction was high in the late stages. Sucrose phosphate synthase (SPS), which is responsible for catalyzing the irreversible reaction of UDP-glucose and fructose-6-phosphate to sucrose-phosphate, exhibited a high level of activity at the early stages (S1–S3) and then declined in the late stages, while SUS activity in the synthetic direction increased with seed development, suggesting its role in sucrose synthesis at late stages, while SPS dominates in early stage (Fig. 7B). Similarly, the activity of starch synthase (SS) and granule-bound starch synthase (GBSS) increased with seed development, which was consistent with the starch content (Fig. 7C and 2H). These results revealed a tight link between sugar metabolism and seed development and suggest that invertase may well play a crucial role in the early stages of seed development.

To reveal how sugar transport and glycometabolism are modulated and integrated at th transcriptional level in seeds, we further screened the key enzyme-encoding genes responsible for sugar metabolism during *C. oleifera* seed development based on the transcriptomic data (sequencing data available via the NCBI BioProject PR-JNA527178) published by Gong et al. [49]. The raw transcriptome data for use in remapping to the *C. oleifera* reference genome (sequencing data available via the NCBI BioProject PRJNA780224 from Gong et al. [40]) were downloaded, and the relative expression levels of key genes related to sugar metabolism, including *CWINs*, *CINs*, *VINs*, *INHs* (invertase inhibitor), *SSs*, *GBSSs*, *SPSs*, and *SUSs* (Fig. 7D), were analysed. For *CWINs*, 11 members were identified and named as *CoCWIN1-CoCWIN11*. Transcriptome data indicated that *CoCWIN9* was highly expressed in the early stage of seed morphogenesis, followed by *CoCWIN11* mainly expressed in the late stage (Fig. 7D). In addition, 13 *CoCIN*, 6 *CoVIN*, 7 *CoINH*, 4 *CoSUS*, 5 *CoSPS*, 3 *CoSS*, and 5 *CoGBSS* genes were identified (Fig. 7D). Among them, *CoCIN1/8*, *CoVIN5*, *CoINH5*, *CoSUS1*, *CoSPS1/4*, *CoSS2*, and *CoGBSS4*, which had the highest expression levels in each family, implying that these genes may be essential for seed glycometabolism.

Since CWIN has been reported to function in conjunction with sugar transporters in regulating sugar allocation and signalling during seed and fruit development [28, 50], we detected the potential action sites of *CoCWINs* in *C. oleifera* seeds. *CoCWIN9* and *CoCWIN11*, which had the higher transcript levels among *CoCWIN* family members, were chosen as the candidates for *in situ* hybridization. The hybridization signals of *CoCWIN9* appeared on the micropylar cellularizing endosperm, transfer cells, and chalazal nucellus at the maternal–filial interfaces (Fig. 7E1, F1, and G1), while *CWIN11* RNA was abundantly expressed at the transfer cells and chalazal nucellus (Fig. 7H1, I1). No signal was observed in sections treated with the sense probes of *CoCWIN9* or *CoCWIN11* (Fig. 7E2, F2, G2, H2, and I2).

## Discussion

Phloem unloading and postphloem transport for photoassimilates in seeds comprise a cohort of transport events that contribute to embryo morphogenesis, starch accumulation, and ultimate yield productivity [34, 51]. The development of seed in relation to sugar transport has so far only been elaborately depicted in the species of Brassicaceae, Fabaceae, and Gramineae [12]. The knowledge of the link between sugar transport and seed development among diverse plant species is very limited. For Gramineae crops such as rice, maize, and wheat, endosperms acting as the final storage domain and seed filling in relation to yield potential have been widely studied [7, 22, 33], nevertheless, for those plants in which the embryo develops into a terminal storage domain accompanied by apoptosis of the endosperm, such as *C. oleifera*, an important woody cash crop with polycarpellary fruits, the nutrient supply, and translocation mechanisms in the seed remain elusive. In this study, we constructed the nexus of nutritional dynamics and seed development and established the molecular model for sugar import into and within *C. oleifera* seeds, which represent a different type of seed development from that of Gramineae crops.

### Dynamic landscape of embryo body development and assimilates accumulation in *C. oleifera*

In *C. oleifera*, ovules (seeds) are inverted into the locules of fruits. The inner integument is closed above the nucellus, and the zygotic embryo, along with the micropyle, lies near the side of the funicle (Fig. 1). As typical dicotyledonous plants, *C. oleifera* seeds accumulate storage products in mature embryos, which determines the final yield of the seeds. According to our observations, it took approximately 3 months for the transformation from the globular to the enlarged cotyledonary shape during seed development in *C. oleifera*. This transformation was accompanied by the development process of endosperm, including nuclear division and cellularization from the micropylar region to the chalazal region, ultimately to its complete disaggregation. We have termed this 3-month period as the “*C. oleifera* seed morphogenesis stage”. In general, embryos that accumulate lipid as a major storage product contain very little starch at maturity, but all oilseeds thus far studied accumulate starch at intermediate stages of development [52]. In *C. oleifera*, during the early stages of seed morphogenesis, small amounts of starch granules were temporarily accumulated first in the seed coats and then in the micropylar endosperm and suspensor; during the late stages of seed morphogenesis, a large amount of starch granules accumulate in endosperm cells and are eventually transferred to the embryo. The maternal seed coat is a major short-term starch storage tissue. Before the endosperm cellularization, sucrose delivered to the seeds is preferentially directed to the seed coat for starch synthesis [53, 54], possibly ensuring the sink strength of the young seeds. In the filial tissues, the preferential accumulation of starch in the micropylar endosperm and suspensor ensured the effective supply of nutrients to embryo in the early stages [55, 56]. As the seeds develop, mature endosperm tissue serves as a temporary storage site for nutrients and surrounds the embryo to support its development [57], and the development and expansion of the embryo in this stage are achieved through the consumption of endosperm. These results suggested that starch accumulation could regulate nutrient allocation between the different tissues of the seed and therefore promote seed development.

### Pathway and entry point for sugar into the developing *C. oleifera* seeds

The starch synthesis in developing seeds depends on a continuous supply of assimilates [53], so we further investigated the transport mechanism by which assimilates are delivered into seeds. In *C. oleifera* fruits, assimilates are required to pass through the carpopodium, axile placentation, and funicle and finally reach the seed (Fig. 3). Microscopy observation revealed that the vascular bundle of *C. oleifera* seed was connected to the funicle and placenta and was limited to only the seed coat. In these maternal tissues, the fluorescence of tracer CF was observed to distribute in the vascular tissue and adjacent cells, suggesting that assimilates translocate through a symplasmic route dependent on plasmodesmata connecting different cells. Nevertheless, the tracer CF was strictly confined to the seed coat and absent in the filial tissues (Fig. 3), suggesting that the symplast isolation between maternal tissues and filial tissues in *C. oleifera* seeds, as has been reported for other plants [8, 31, 58]. Our results provide real-time evidence for the apoplasmic route of postphloem transport from the maternal to the filial tissues during *C. oleifera* seed development, while the symplasmic connections between the maternal tissues ensure the sufficient input of assimilates to sink tissues.

Various sugar transporters function at the apoplasmic step, determining seed development and yield [33, 59]. In this study, four sugar transporters with high mRNA expression at different stages of seed development were screened: CoSWEET1b, CoSWEET2a, CoSWEET15, and CoSUT2. CoSWEET1b is located on the plasma membrane and can transport glucose and fructose, similarly to members of clade I, such as AtSWEET1 [60]. CoSWEET2a is situated on the vacuolar membrane, similar to its homologous protein AtSWEET2 [61], and is able to transport sucrose, glucose, and fructose. CoSWEET15 is localized on the plasma membrane and has the ability of transporting sucrose, comparable with clade III members such as OsSWEET15 [7] and AtSWEET15 [62]. CoSUT2 is localized on the plasma membrane and is involved in sucrose uptake, much like its homologues AtSUT2 [16] and AcSUT2 [63]. We further determined the ability of these proteins to transport sugars and respond to sugar signals in transgenic calli and *C. oleifera* seeds. All of these proteins can be induced by sucrose, glucose, and fructose and promote soluble sugar accumulation and calli growth (Fig. 5), further demonstrating that the four transporters can be induced by sugar signals and play a role in facilitating sugar transport. On the basis of the acting sites of these proteins, combined with the complete morphological characteristics of the seeds, we propose three entry sites for nutrients into the endosperm or embryo in *C. oleifera* seeds:

(1) The first site is the chalazal nucellus, where the mRNAs of *CoSWEET1b* and *CoSUT2* are located. Chalazal nucellus sits between the endosperm and chalaza and has been widely reported as a key entry point for developing seeds in other plant species [7, 64]. Lu’s study showed that the nucellus is symplasmically connected to the chalaza, the nutrient unloading zone in seed, and acts as sugar source alongside the seed coat in *Arabidoposis* [65]. Similar to its homologues in barley [66], rice [64], and wheat [67], CoSUT2, a sucrose intake transporter, is also expressed in the nucellus dedicated to sugar efflux and is likely to function in a mechanism of solute retrieval from the apoplast and maintain an intra- or extra-cellular sucrose equilibrium [12].

(2) The second entry site is transfer cells, where the mRNAs of *CoSWEET1b*, *CoSWEET2a*, and *CoSUT2* are located. Transfer cells are specialized for membrane transport of nutrients and are usually located on one or both sides of the maternal/filial interface in the developing seeds of many plant species [68]. It is noteworthy that there is a significant difference in seed structure between *C. oleifera* and cereal. *C. oleifera* seeds lack aleurone and have only a single layer of transfer cell, where sugar transporters are located, to connect to the seed coat and the endosperm. This difference indicates that the transfer cells may have a dual function in absorbing sugar from the seed coat and expelling it into the endosperm. In cotton, the transfer cells that encapsulate the endosperm play a major role in nutrient transport during seed development [29]. Transfer cells have evolved a great potential capacity to transport sucrose and conferred an evolutionary increase in seed and fibre biomass yield [68]. Future studies on the function of these transporters may reveal the main entry point of transfer cells for sugar in bulk into the filial tissue in *C. oleifera*, and it necessitates exploring the origin and fate of transfer cells in *C. oleifera* by excavating genes expressed in transfer cells.

(3) The third site is the micropylar cellularizing endosperm, where *CoSWEET15* mRNA is located. The micropylar endosperm is adjacent to the micropylar end of the embryo sac and suspensor and is formed earlier than the chalazal endosperm [69]. The preferential development of micropylar endosperm increased the number of apoplasmic junctions connecting maternal tissues with the embryo proper [5]. In the early stages of *C. oleifera* seed development, starch preferentially accumulates in the suspensor and micropylar endosperm, where *CoSWEET15* transcripts are specifically localized (Fig. 2B and Fig. 7D) and the transcript signal of *CoSWEET15* cannot be detected with the disappearance of suspensor (Fig. S10). Therefore, the seed coat-micropylar endosperm/suspensor-embryo is articulated as the channel in the early stages of embryo development through which assimilates are transported to the embryo. Meanwhile, *CoSWEET15* functions as a micropylar endosperm specific gene in the early stages of embryo differentiation dependent on a suspensor to transfer nutrients from the seed coat to embryo.

In conclusion, the three entry sites—the chalazal nucellus, micropylar cellularizing endosperm, and transfer cells at the interfaces between maternal and filial tissues constitute an efficient ‘gateway’ channel for postphloem transport of sugar regulated by various sugar transporters during *C. oleifera* seed development. Sucrose was reported to be the main form of sugar transported in *C. oleifera* leaves, and phloem unloading of sugars in the fruit pericarp was shown to occur through the transition from a symplasmic to an apoplasmic pattern [41, 51]. Combined with these findings, we construct a model of multistep sequential sym-apoplasmic transport processes from carpopodium to embryo in *C. oleifera* fruit (Supplementary Fig. S11), providing new knowledge for revealing the polycarpellary fruit development mechanism in plants.

### Pattern of sugar utilization in the developing *C. oleifera* seeds

Sugar transport needs to be coupled with sugar metabolism for growth and development [50]. In the early stages of *C. oleifera* embryo morphogenesis, a high hexose-to-sucrose ratio is mediated by high-activity CWIN and VIN, which stimulate mitotic activity to increase cell number in developing seeds [7]. In late stages, sucrose replaced the hexose as the major sugar in seeds, and the content of starch increased rapidly. High-content sucrose acts as a differentiation signal to trigger the accumulation of storage products, thus affecting seed development [70]. During the late stage, the high sucrose content was maintained by the high-activity SUS enzyme in the synthesis direction. High activity of SUS enzyme in the cleavage direction can provide more UDP-Glu for starch synthesis. SPS activity followed a similar pattern to CWIN activity, suggesting that this pair of enzymes is mainly responsible for the sucrose metabolism balance in the early stages. These results also revealed the tight link between sugar metabolism and seed development. The high expression of *CoCWIN9*, *CoCIN1*, *CoVIN5*, *CoSUS1*, *CoSPS1/4*, *CoSS2*, and *CoGBSS4* accentuates their potential roles in the regulation of sucrose metabolism and seed development. Considering that CoSUT2 was highly expressed in the embryo, especially in the late stages (Fig. 4), we cannot exclude the possibility that CoSUT2 plays a major role in maintaining high levels of sucrose in the expanding embryo during the late stages of *C. oleifera* seeds and cooperates with CoSUS1 to facilitate starch synthesis and oil accumulation.

### A model of how CWIN-coupled sugar transporters control sucrose entry into the *C. oleifera* seeds

**Figure 8:**
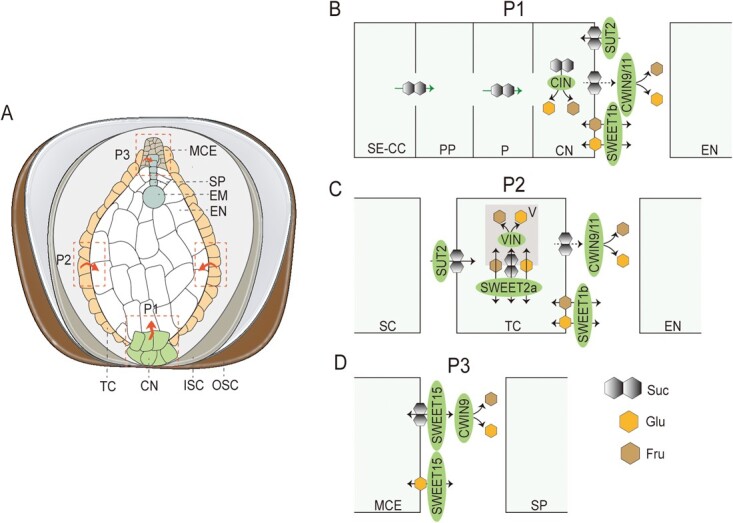
A hypothetical model for molecular regulation of sugar import into and within *C. oleifera* seeds. (A) Sucrose is apoplasmically unloaded from the phloem and transported from maternal tissues to filial tissues via three pathways in *C. oleifera* seeds. The pathway 1 (P1) is via chalazal nucellus to endosperm, the pathway 2 (P2) is via transfer cells to endosperm, and the pathway 3 (P3) is via micropylar endosperm to suspensor. (B) In the pathway 1, CoSWEET1b is responsible for export of glucose and fructose from chalazal nucellus into apoplast, where the sucrose can be hydrolyzed into glucose and fructose by CoCWIN9 and 11. CoSUT2 is responsible for absorption of sucrose. (C) In the pathway 2, sucrose moves toward and into the transfer cells mediated by CoSUT2. CoSWEET1b is responsible for export of glucose and fructose from the transfer cells into the apoplast near the endosperm, where the sucrose can be further hydrolyzed into glucose and fructose by CoCWIN9 and 11. (D) pathway 3, CoSWEET15 is responsible for the transfer of sucrose and glucose from the micropylar cellularizing endosperm cells into the apoplasmic space, coupled with CoCWIN9 hydrolyzing sucrose into glucose and fructose. In the diagram, green arrows represent that sugars are transported by plasmodesmata, black arrows represent that sugars are transported through sugar transporters, and the black dotted arrows stands for unknown transporters that are responsible for sucrose export. P1, pathway 1; P2, pathway 2; P3, pathway 3; CWINs, cell wall invertases; MCE, micropylar cellularizing endosperm; SP, suspensor; EM, embryo; EN, endosperm; TC, transfer cells; CN, chalazal nucellar; ISC, inner seed coat; OSC, outer seed coat; SE-CC, sieve element/companion cell complex; PP, phloem parenchymal cell; P, parenchymal cell; SC, seed coat; V, vacuole; Suc, sucrose; Glu, glucose; Fru, fructose.

During the apoplasmic step of phloem or postphloem unloading, sucrose is either directly mediated by sucrose transporters or initially hydrolyzed into hexose by CWINs and subsequently imported into sink cells through hexose transporters [71]. Here, *CoCWIN9* and *CoCWIN11* were highly transcribed and localized in micropylar cellularizing endosperm, transfer cells, and chalazal nucellus, where sugar transporter signals were also detected. Based on spatiotemporal expression of these proteins and in conjunction with pathways for sugar transport and metabolism, we propose a model of sugar transporter-mediated and CWIN-coupled sucrose entry from seed coat into embryo in *C. oleifera* seeds (Fig. 8). (i) Sucrose is transported from the seed coat to the chalazal nucellus through symplasmic route. In chalazal nucellus, glucose and fructose which are produced by sugar metabolism can be transported by CoSWEET1b to the apoplast adjacent to the chalazal endosperm. Sucrose in apoplast can be retrieved by CoSUT2. (ii) The CoSUT2 protein localized in the transfer cells may mediate the absorption of sucrose, while CoSWEET1b is responsible for the efflux of glucose and fructose from the transfer cells to the apoplasmic space adjacent to the endosperm. Cytosolic sugar homeostasis in the transfer cells is regulated by the balance between the transport of sugars across tonoplast, possibly mediated by CoSWEET2a. For pathways 1 (P1) and 2 (P2), sucrose in the apoplast can be hydrolyzed by CoCWIN9 and CoCWIN11, providing more hexoses to liquid endosperm for nuclear division [29]. (iii) CoSWEET15 is responsible for the transport of sucrose and glucose from the exit of the micropylar endosperm to the apoplasmic space adjacent to the suspensor in the early stage of seed development, while a portion of the sucrose is hydrolyzed into hexose by CoCWIN9 and the imported sugars eventually reach the embryo via the suspensor.

Sugar transporters have been reported to be localized in the embryo in some plant species. For instance, the embryo-localized ZmSUT4 facilitates sucrose uptake in the embryo of maize [8]. In cotton, the *GhCWIN1* transcript was detected in globular embryos [29]. In our study, *CoSWEET15* and *CoSUT2* showed high expression in the embryo, whereas no mRNA signals of sugar transporters or CWIN were detected by *in situ* hybridization in the embryo of *C. oleifera*. Thus, the transfer of nutrients from the endosperm to the embryo after the disappearance of the suspensor in *C. oleifera* needs further investigation in the future to explore what proteins are responsible for sugar import into the embryo in the future. In addition, it is also necessary to obtain a knockout mutant of CoSWEET15 to explore its function in the early differentiation of embryo and relation to suspensor. In summary, our findings provide the first holistic evidence of the spatiotemporal control of sugar transport and partitioning regulated by sugar transporters and CWINs in *C. oleifera* seeds, as well as new potential targets for the improvement of seed yield.

## Materials and methods

### Plant materials

*Camellia oleifera* var. ‘Hua Shuo’ plants grown in the Wangcheng District, Changsha City, Hunan Province, were used for sampling developing seeds at various stages. After pollination which began early in November, the seeds were collected regularly from 180 to 280 DAP. The sampled seeds were divided into six developmental stages: stage 1 (S1, 200 DAP), stage 2 (S2, 220 DAP), stage 3 (S3, 240 DAP), stage 4 (S4, 260 DAP), stage 5 (S5, 280 DAP), and stage 6 (S6, 280 DAP). Half of the fresh samples were used for the paraffin sectioning, and the other half were immediately frozen in liquid nitrogen and subsequently stored at −80°C.

### Paraffin sections

The anatomic structure of the fruits and seeds was observed according to Du’s description [72], with minor modifications. First, complete fruits and the separated seeds were collected and fixed immediately in Carnoy’s solution (a 3:1 formaldehyde–ethanol mixture). After 24 h, the samples were dehydrated with graded ethanol and infiltrated with a xylene/ethanol series, which were slowly permeated overnight with xylene/paraffin series. Then, the treated samples were embedded in a wax solution. When the wax chunks cooled and solidified, the samples were cut into 8 μm thick slices by using a rotary microtome (Leica RM2265, Berlin, Germany). Following deparaffinizing and hydrating with a xylene/ethanol series, the tissue sections were observed under an upright fluorescence microscope (Leica DM 2500, Berlin, Germany).

### Starch staining of seed sections

The tissue sections of seeds at different stages were stained with 0.1% aqueous I_2_-KI for 10 min as described by Zhu *et al*. [73] and washed with deionized water, after which the starch granules were observed and photographed under a biological microscope (Leica DM 2500, Berlin, Germany).

### CF diacetate labelling

Carboxyfluorescein diacetate (CFDA; Sigma Aldrich) (1 mg/ml) was introduced into the fruits and seeds at 20 DAP and 220 DAP, respectively, from the peduncle as described in Zhang *et al*. [43, 74]. When CFDA enters live cells, it decomposes via the removal of the acetate groups at the 3′ and 6′ positions with the help of intracellular esterases, and the fluorescent and membrane-impermeable CF is thus released. On a sunny day, a needle with thread was passed through a centrifuge tube containing 150 μl of CFDA and an absorbent cotton ball, and then impaled into the phloem of the peduncle. The CFDA was slowly drained to the phloem by the thread. After 48 h, the fruits were sampled and immediately sliced freehand into transverse and longitudinal sections. Tissue sections were observed under a Leica SP8 confocal laser-scanning microscope using the GFP detection channel (488 nm).

### RNA extraction and gene expression analysis

RNA extraction and qRT-PCR procedures were carried out using the methodology described by Zhou *et al*. [75]. qRT-PCR was employed to assess the expression levels of *CoSWEET1b*, *CoSWEET2a*, *CoSWEET15*, and *CoSUT2* genes across different tissues (roots, stems, spires, mature leaves, flowers, seeds of different stages, embryos, endosperm, seed coats, ovules cultured *in vitro* and calli) and developmental stages of *C. oleifera* seeds. The obtained data were log_2_ transformed, and heatmaps were generated using TBtool software for visualization. The relative expression of the genes was calculated by the 2^−∆∆CT^ method. All the experiments were repeated three times by using independently prepared RNA/cDNA templates.

### Subcellular localization in *N. benthamiana* pavement cells and protoplasts

The CDSs of *CoSWEET1b*, *CoSWEET2a*, *CoSWEET15*, and *CoSUT2* without stop codons were constructed separately on the pCAMBIA super 1300 vector to form C-terminal fusions with the GFP reporter under the control of the CaMV35S promoter. The sequences of all primers used are listed in Supplementary Table S2. The pCAMBIA1300-CoSWEET1b-GFP, pCAMBIA1300-CoSWEET2a-GFP, and pCAMBIA1300-CoSUT2-GFP were co-transformed with pBI121-mCherry-fABD2 (plasma membrane marker [76] into *N. benthamiana* (tobacco) pavement cells. pCAMBIA1300-CoSWEET2a-GFP and vac-CD3-971 (the vacuolar membrane marker) [77] were co-transformed into tobacco pavement cells. The empty vector expressing untargeted GFP was used as a control. The fluorescence signals were observed under a confocal laser microscope (Carl Zeiss LSM780) with GFP (excitation and emission wavelengths of 440 and 500 nm) and RFP (excitation and emission wavelengths of 540 and 600 nm), respectively. The fluorescence of GFP and mCherry was measured by using Image-Pro Plus 6.0 software.

For protoplast localization, pCAMBIA1300-CoSWEET2a-GFP and Atγ-TIP (the vacuolar membrane marker) [78] were cotransformed into tobacco pavement cells. After 48 h, the protoplasts were extracted from the infected leaves according to the protocol previously described [79]. The fluorescence in the protoplasts was detected under the laser confocal microscope.

### Subcellular localization in yeast

For the subcellular localization of CoSWEET2a in yeast, CoSWEET2a-GFP fusion fragments were cloned into the yeast expression vector pDR196, following previously described methods [80]. The construct was transformed into the EBY.VW4000 yeast strains, which were cultured on SC/-Ura plates supplemented with glucose. After 3 days, the yeast cells were re-suspended and observed under a Leica confocal microscope at wavelengths of 488 and 560 nm for excitation and emission, respectively.

### Complementation of the yeast mutant

The CDSs of *AtSUC2*, *SiSWEET7a*, *CoSWEET1b*, *CoSWEET2a*, *CoSWEET15*, and *CoSUT2* were inserted into the vector pDR196. Each fusion vector was separately transformed into the yeast (*S. cerevisiae*) mutant strains SUSY7/ura3, which is deficient in sucrose transport, or EBY.VW4000, which is deficient in hexose transport, together with the empty vector (negative control), by using the lithium acetate method [81]. The sequences of all primers used are listed in Supplementary Table S2. The recombinant vectors pDR196-AtSUC2 and pDR196-SlSWEET7a were used as positive controls for EBY.VW4000 and SUSY7/ura3, respectively [82, 83]. The EBY.VW4000-positive clones were cultured on SC/-Ura plates supplemented with 2% (w/v) glucose or fructose as the sole carbon source, and SUSY7/ura3-positive clones were cultured on SC/-Ura plates supplemented with 2% (w/v) sucrose as the sole carbon source to examine yeast growth at different concentrations at 30°C for 3 d.

### Glucose uptake assay using fluorescent 2-NBDG in the yeast system

The 2-NBDG uptake assay was mainly performed as described previously, with minor modifications [48]. In brief, the EBY.VW4000 yeast cells of pDR196 vector, CoSWEET2a-pDR196, and SlSWEET7a-pDR196 were initially cultured in YPDA with maltose. SlSWEET7a were used as a positive control. Then, the cells were harvested and re-suspended with 60 μM of 2-NBDG (Sigma). Finally, the cells were visualized with a Leica confocal microscope using the GFP filter with the excitation wavelengths of 488 nm.

### Sucrose transporter assay using fluorescent esculin substrate

The esculin uptake assay was mainly performed, as described previously [48]. In brief, the SUSY7/ura3 yeast cells of pDR196 vector, CoSWEET2a-pDR196 and AtSUC2-pDR196 were initially cultured in YPDA with glucose. AtSUC2 was used as a positive control. Then, the cells were harvested by centrifugation, and re-suspended in the Na_2_HPO_4_ at pH 4.0 with 1 mM esculin (Sigma). After being cultured for 1 h, the cells were observed under a Leica confocal microscope using the excitation wavelengths of 420–460 nm.

### *C. oleifera* seeds *in vitro* culture on acarbose medium

This experiment was performed based on the methods of Sosso *et al*. [22] with modifications. *C. oleifera* seeds in S3 were sterilized with 75% alcohol for 45 s and subsequently sterilized with 1% corrosive sublimate for 10 min. Approximately 10 seeds were placed on various MS solid medium supplemented with 3% sucrose, 3% glucose, and 3% fructose in the funicle-down direction, and incubated in a dark growth chamber at 24°C for 8 days. MS solid medium without sugar was used as a control. All medium supplemented with 1 mg/l 2,4-Dichlorophenoxyacetic acid and 10 mg/l streptomycin sulfate at a pH of 5.8. Total RNA was extracted from all the samples as described above.

### Transformation of apple calli

The CDSs of *CoSWEET1b*, *CoSWEET2a*, *CoSWEET15*, and *CoSUT2* without stop codons were constructed separately on the pCAMBIA 1300 vector containing a GFP tag, followed by the transformation of *Agrobacterium tumefaciens* strain GV3101. The genetic transformation method was performed as previously described by Wang *et al*. [84]. In the dark, the apple calli that had grown for 15 days were infected with the *Agrobacterium* for 30 min, and cocultured on MS solid medium without antibiotics at 24°C for 48 h. Then, the calli were transferred to a selection medium supplemented with kanamycin and carbenicillin. After 10 days, the expression of CoSWEET1b, CoSWEET2a, CoSWEET15, and CoSUT2 in the calli was confirmed by GFP fluorescence observation under a stereomicroscope via the GFP detection channel and PCR amplification. Finally, the transgenic calli were, respectively, placed on various MS solid medium supplemented with 3% sucrose, 3% glucose, and 3% fructose and incubated in the dark at 24°C for 15 days. MS solid medium without sugar was used as a control.

### *In situ* hybridization

The process of *in situ* hybridization was carried out according to the previously described method [8, 29, 85]. The gene-specific probes used for *CoSWEET1b*, *CoSWEET2a*, *CoSWEET15*, *CoSUT2*, *CoCWIN9*, and *CoCWIN11* were listed in Supplementary Table S3. All sense and antisense RNA transcripts were synthesized by T3 and T7 RNA polymerase with digoxigenin-UTP (from Roche Diagnostics) as the label. Tissue sections of *C. oleifera* seeds were obtained using the paraffin sectioning method as described above. Briefly, dewaxed tissue sections were sequentially treated with 10 μg/ml proteinase K, 0.2% (w/v) glycine, 4% (w/v) paraformaldehyde, and 0.5% (w/v) acetic anhydride. Then, the tissue sections were incubated with hybridization mixture overnight at 50°C in a humidified chamber. The hybridization mixture was combined with 500 μl of *in situ* hybridization salts (800 mM NaCl, 100 mM Tris–HCl, 100 mM sodium phosphate, and 50 mM EDTA), 2 ml of deionized formamide, 1 ml of 50% (w/v) dextran sulfate, 100 μl of 50 × Denhardt’s solution, 50 μl of 100 mg/ml tRNA, 350 μl of diethylpyrocarbonate (DEPC) H_2_O, and 6 μg of RNA probe. After 16-20 h, the slides were sequentially washed in 0.2 × SSC buffer (pH 7.0), NTE buffer and TBS buffer and then incubated with 10 × Roche blocking solution. The hybridized probes were detected using an alkaline phosphatase-conjugated anti-DIG (digoxigenin from Roche) antibody and NBT/BCIP (from Roche) as substrates. The positive signals representing hybridized RNAs in localized tissues were photographed by using a biological microscope (Leica DM 2500, Berlin, Germany).

### Physiological measurements

The determination of the total soluble sugar, glucose, fructose, sucrose, and starch contents was conducted based on previous methods [38, 86]. The carbohydrates from the freeze-dried samples (0.1 g) were extracted with 80% ethanol at 80°C for 10 min, after which the total soluble sugar, glucose, fructose, and sucrose contents in the supernatants were determined. The sediment was hydrolysed with 30% HCl for 15 min, followed by 9.5 mol/L NaOH to neutralize the acid, after which the supernatants were centrifuged to determine the starch contents. The enzyme activities of CWIN, VIN, CIN, GBSS, SSS, SUS, and SPS in *C. oleifera* seeds were determined according to Jin *et al*. [86] and were normalized on a protein basis.

### Statistical analysis

All the experimental data are presented as the mean values of at least three independent replicates for the biological experiments. The experimental data were subjected to one-way ANOVA and Student’s *t* test in SPSS 22.0.

## Supplementary Material

Web_Material_uhae133

## Date availability

All relevant data in this study can be found in the article and its supporting files.
