# Supplementary material for: Sugar import mediated by sugar transporters and cell wall invertases for seed development in Camellia oleifera
Source: Hortic Res. 2024 May 10;11(7):uhae133. doi: 10.1093/hr/uhae133 (PMC11226869; doi:10.1093/hr/uhae133)
Supplement: Web_Material_uhae133 [file web_material_uhae133.zip › HR-2024-048_Revised supplementary materials.docx]

**Sugar import mediated by sugar transporters and cell wall invertases for seed development in *Camellia oleifera***

Bingshuai Du^1^, Yibo Cao^1^, Jing Zhou^1^, Yuqing Chen^1^, Zhihua Ye^1^, Yiming Huang^1^, Xinyan Zhao^1^, Xinhui Zou^1^, Lingyun Zhang^1*^

^1^State Key Laboratory of Efficient Production of Forest Resources, Key Laboratory of Forest Silviculture and Conservation of the Ministry of Education, The College of Forestry, Beijing Forestry University, Beijing 100083, China

^*^Correspondence: lyzhang@bjfu.edu.cn; Tel.: +86-10-62336044; Fax: +86-10-62338197

**Supplemental Data**

**Supplemental Figure S1.** Dynamic changes of *C. oleifera* fruits with development.

**Supplemental Figure S2.** Hexose-to-sucrose ratio following seed development.

**Supplemental Figure S3.** The transverse sections of the fruits, showing the distribution of locules and seeds.

**Supplemental Figure S4.** Neighbour-joining phylogenetic tree of sugar transporters in *C. oleifera* and *Arabidopsis*.

**Supplemental Figure S5.** The expression profiles of *CoSTPs* during seed morphogenesis.

**Supplemental Figure S6.** Tissue-specific expression of *CoSWEET1b*/*2a*/*15* and *CoSUT2* in flowers, stems, spires, mature leaves, roots and seeds of *C. oleifera*.

**Supplemental Figure S7.** The subcellular localization of CoSWEET2a in yeast.

**Supplemental Figure S8.** The transport activity of CoSWEET2a in yeast using 2-NBDG and esculin.

**Supplemental Figure S9.** Identification of overexpression lines of *CoSWEET1b*/*2a*/*15* and *CoSUT2* in apple calli.

**Supplemental Figure S10.** In situ hybridizations for *CoSWEET15* in 240 DAP seeds.

**Supplemental Figure S11.** Model for multistep sequential symplast-apoplasmic transport steps from carpopodium to embryo in *C. oleifera* fruit.

**Supplementary Table 1.** The CDS sequences of *CoSWEET1b/2a/15*, *CoSUT2*, *CoCWIN9* and *CoCWIN11*.

**Supplementary Table 2.** List of all primers used for the study.

**Supplementary Table 3.** The specific gene fragments in *CoSWEET1b/2a/15*, *CoSUT2*, *CoCWIN9* and *CoCWIN11*.


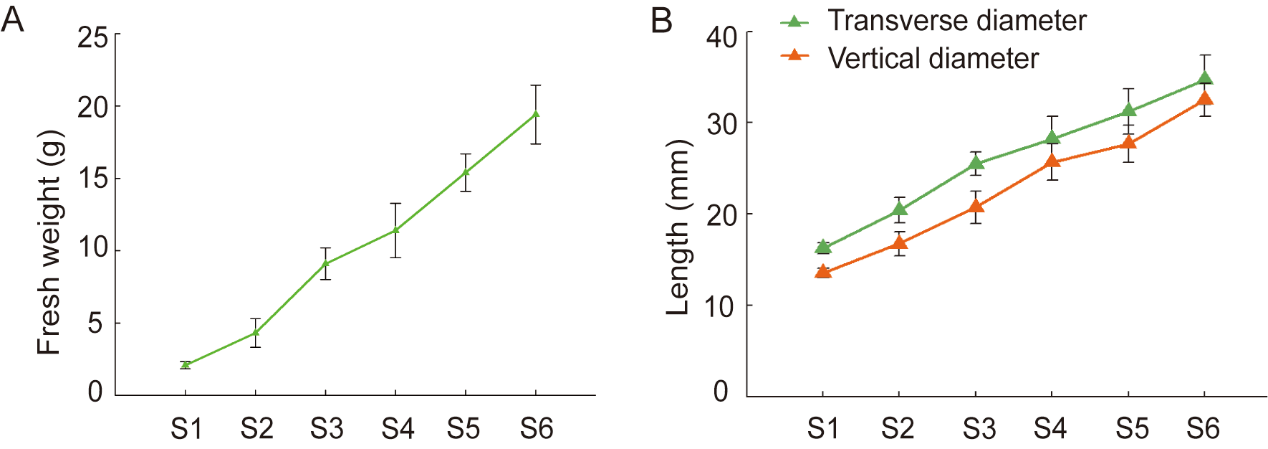


**Figure S1.** Dynamic changes of *C. oleifera* fruits with development. (**A**, **B**) Fruits growth dynamics observation, including fresh weight, transverse diameter, and vertical diameter at different stages.


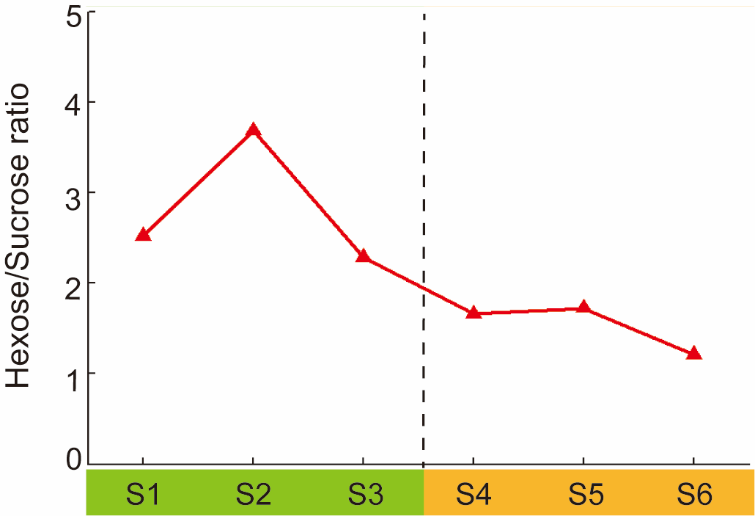


**Figure S2.** Hexose-to-sucrose ratio following seed development. During the seed morphogenesis, the hexose-to-sucrose ratio undergoes the process of first rising and then falling.


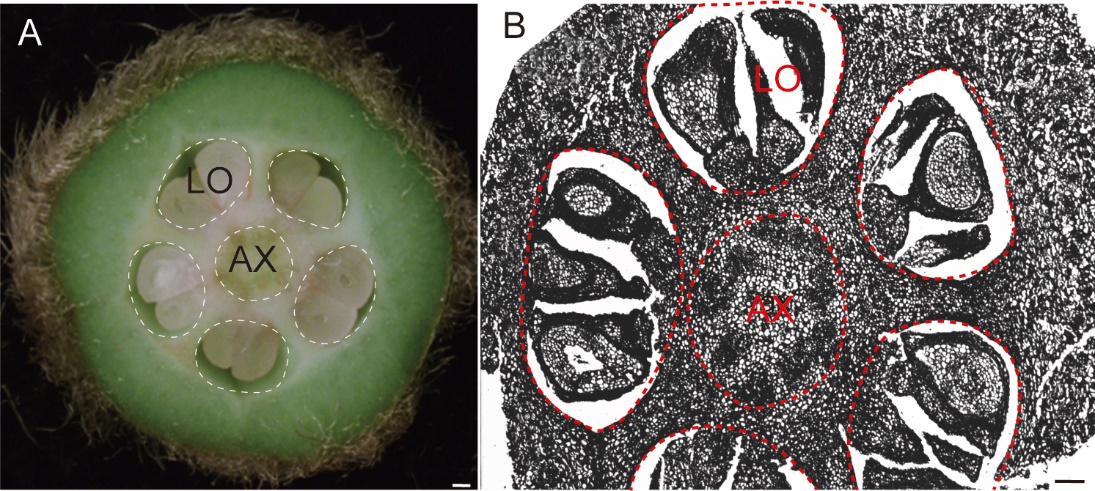


**Figure S3.** The transverse sections of the fruits, showing the distribution of carpels and seeds. There are ~5 carpels that closed toward and connected with the fruit central column to form axile placentation. (**A**) Transverse sections of the fresh fruits. (**B**) Transverse sections of the fruits by the method of paraffin microtomy. LO, locules; AX, axile. The scale bar is 200 μm.


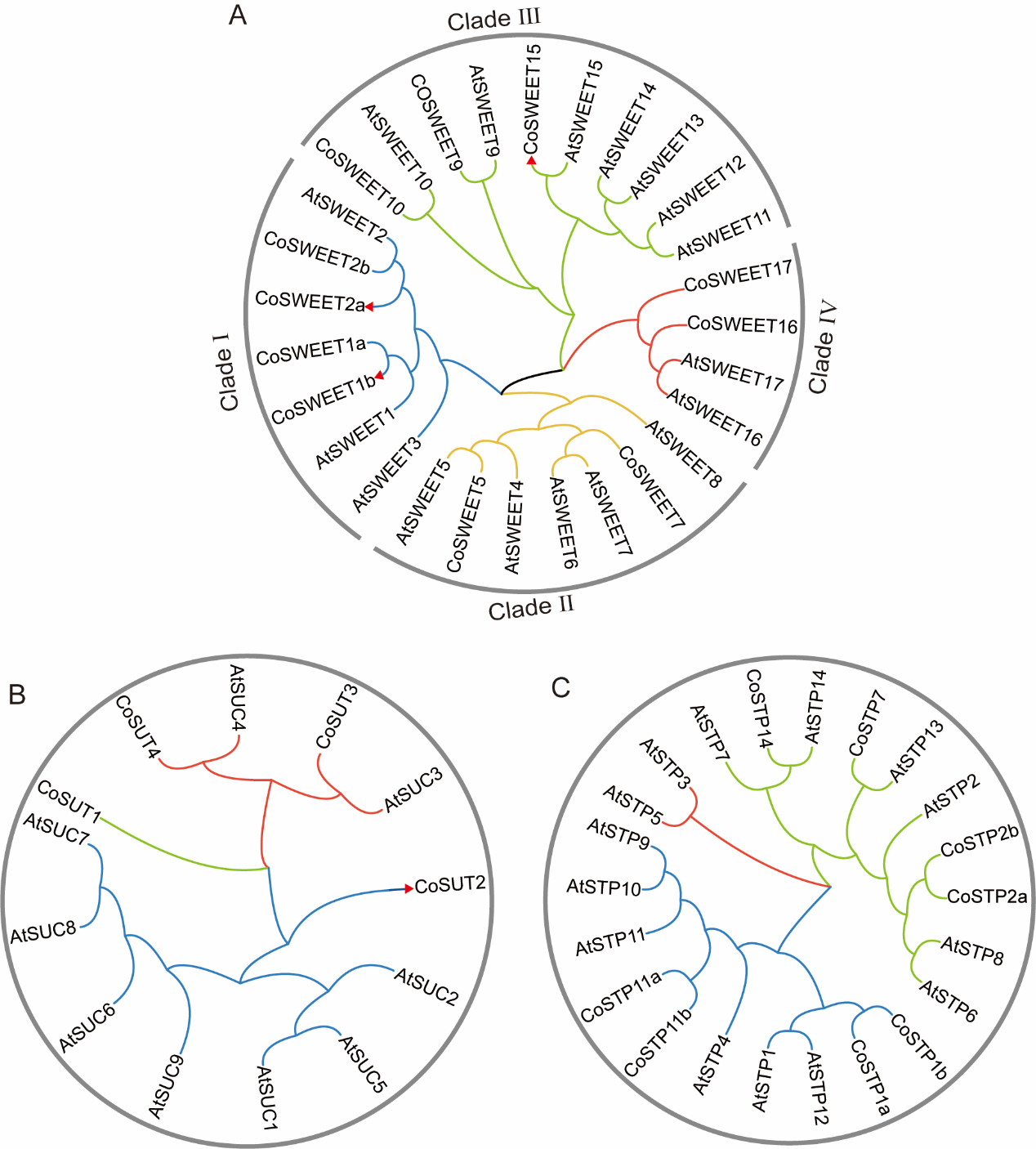


**Figure S4.** Neighbour-joining phylogenetic tree of sugar transporters in *C. oleifera* and *Arabidopsis*. (**A**) Neighbour-joining phylogenetic tree of SWEETs. (**B**) Neighbour-joining phylogenetic tree of SUTs. (**C**) Neighbour-joining phylogenetic tree of STPs.


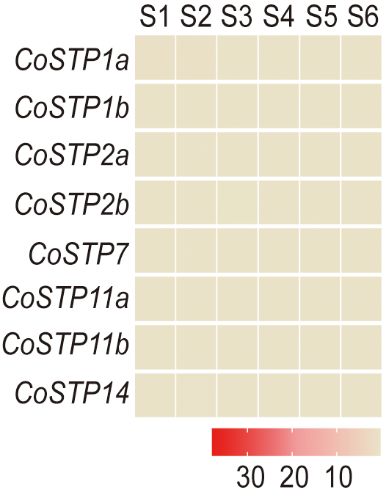


**Figure S5.** The expression profiles of CoSTP transporters during seed morphogenesis. The transcript levels of all of the *CoSTP* genes detected remained low from stage 1 to stage 6.


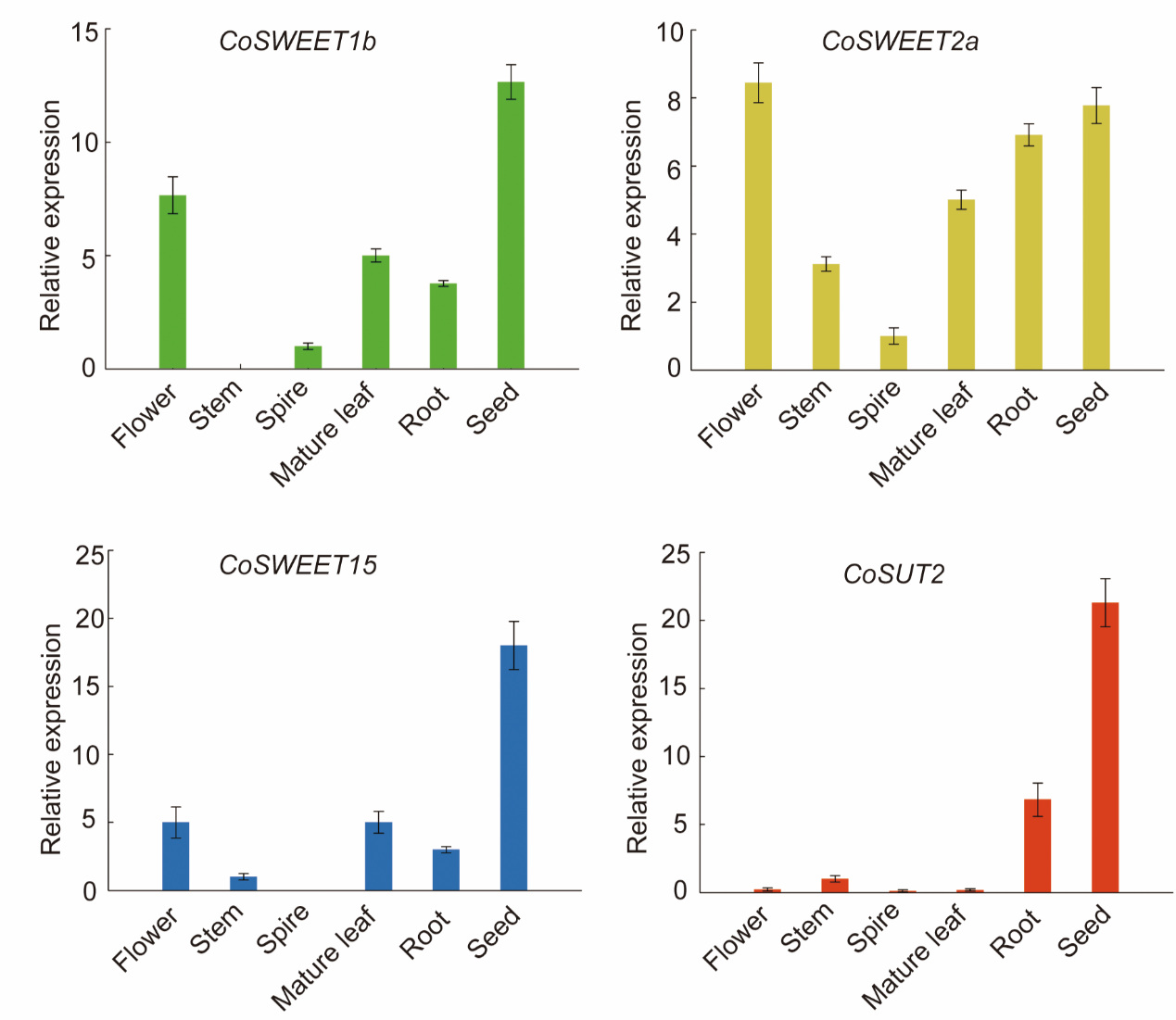


**Figure S6.** Tissue-specific expression of *CoSWEET1b*/*2a*/*15* and *CoSUT2* in flowers, stems, spires, mature leaves, roots and seeds of *C. oleifera*. *CoSWEET1b*, *CoSWEET15* and *CoSUT2* had higher mRNA levels in seeds than other tissues, while *CoSWEET2a* had high expression in flowers, seeds and roots.


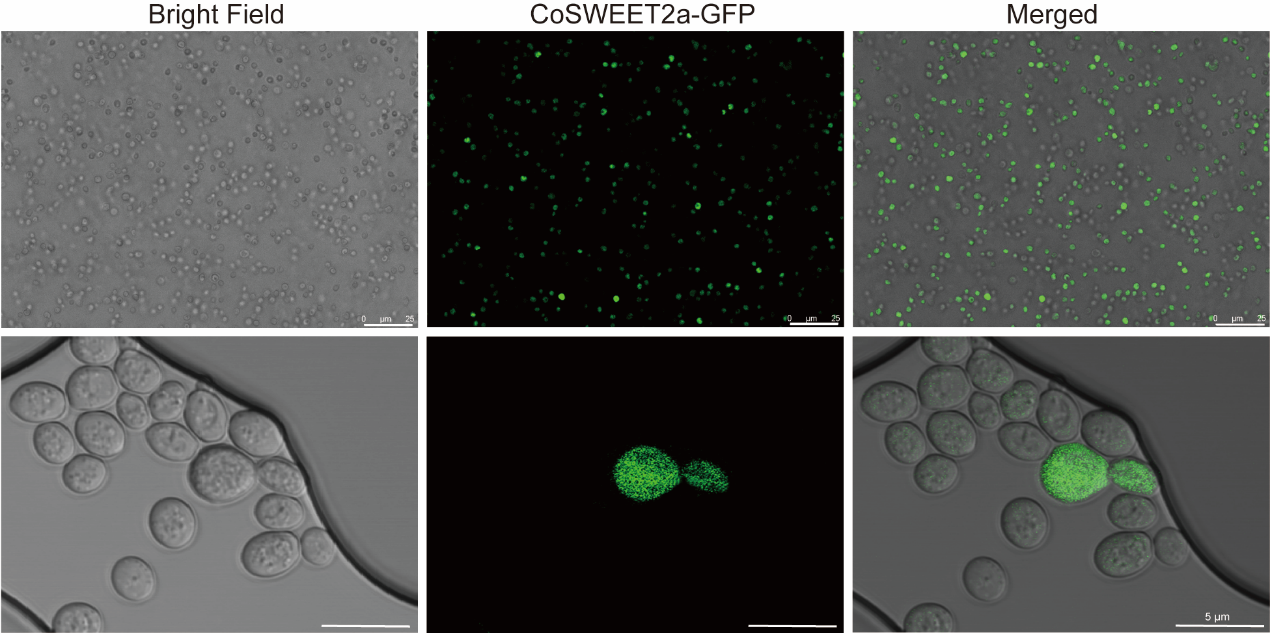


**Figure S7**. The subcellular localization of CoSWEET2a in yeast. The CoSWEET2a-GFP-pDR196 fusion vector was transferred into the mutant yeast strain EBY.VW4000. The results showed that CoSWEET2a could be expressed in yeast mutant cells and was not confined to the yeast’s tonoplast.


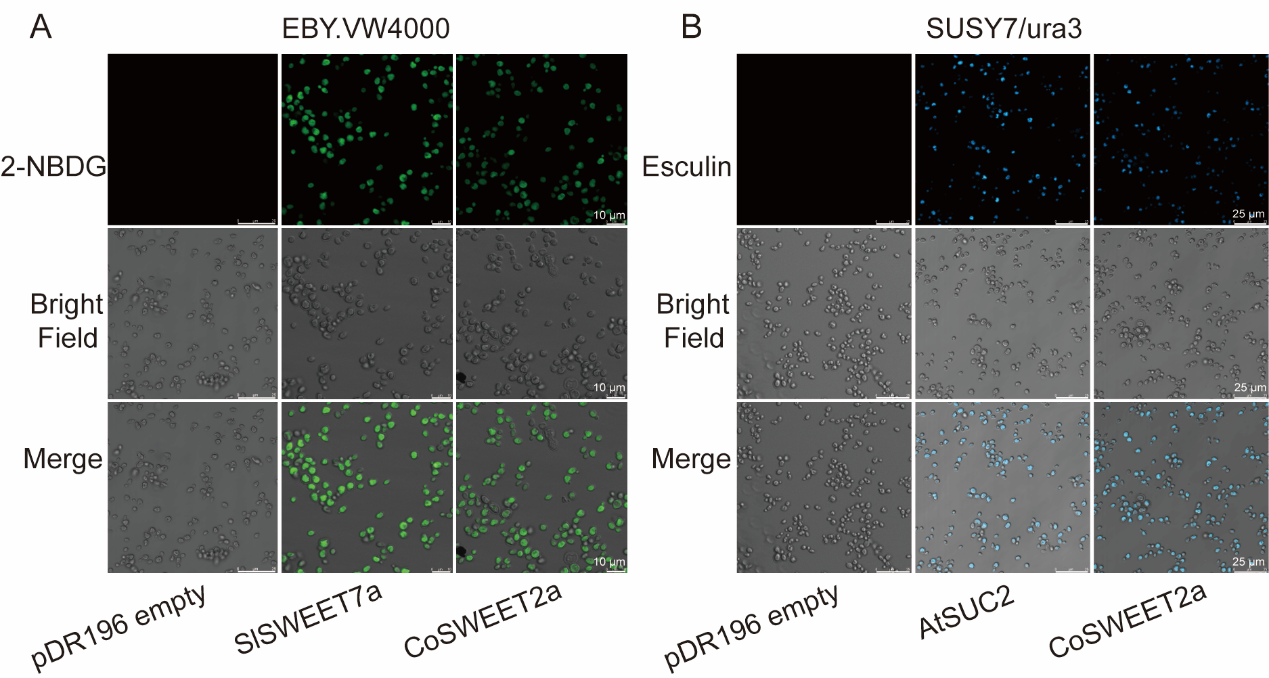


**Figure S8.** The transport activity of CoSWEET2a in yeast based on 2-NBDG and esculin. (A) Uptake of 2-NBDG fluorescent derivative in yeast strain EBY.VW4000 that expressed pDR196 empty and CoSWEET2a, respectively. Yeast mutant strains transformed with SlSWEET7a were used as a positive control. (B) Uptake of the sucrose analogue esculin in yeast strain Susy7/ura3 expressing pDR196 empty and CoSWEET2a, respectively. Yeast mutant strains transformed with AtSUC2 were used as a positive control.


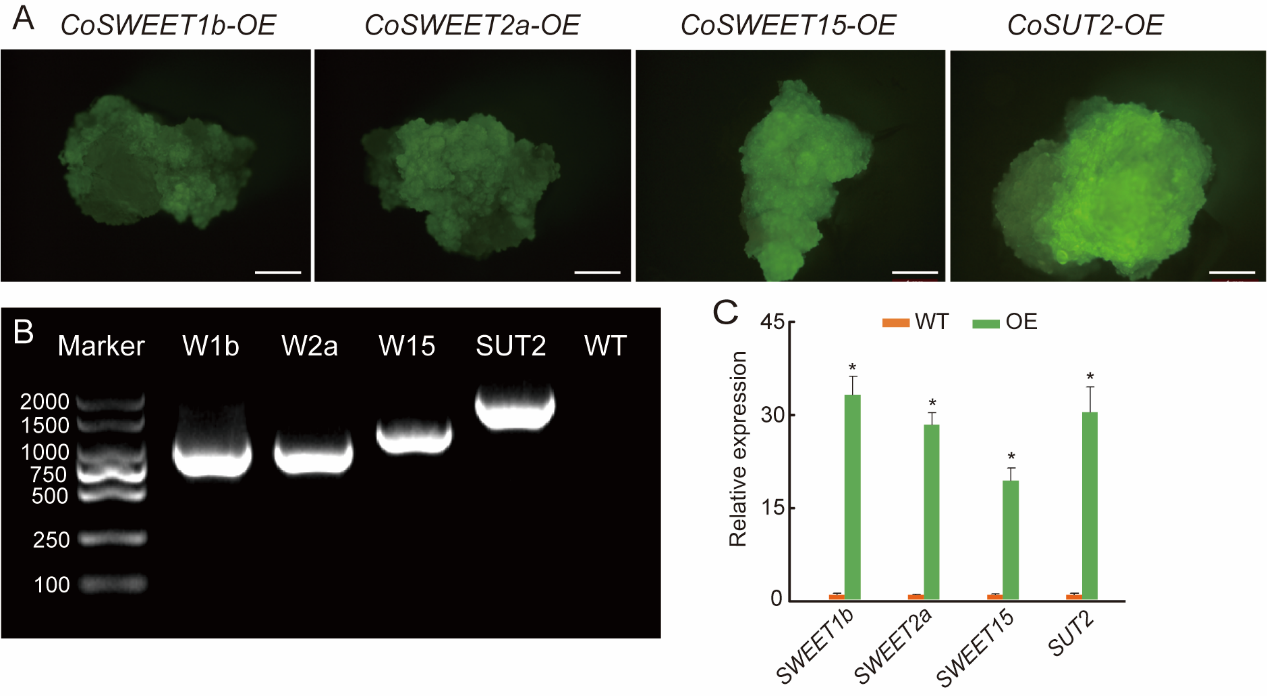
**Figure S9.** Identification of overexpression lines of *CoSWEET1b*/*2a*/*15* and *CoSUT2* in apple callus. (**A**) Identification of overexpression lines of *CoSWEET1b*/*2a*/*15* and *CoSUT2* in apple callus by GFP fluorescence observation. The strong green fluorescence can be detected under the stereo fluorescence microscope. (**B**) Identification of overexpression lines of *CoSWEET1b*/*2a*/*15* and *CoSUT2* in apple callus by PCR. (**C**) Relative expression levels of *CoSWEET1b*, *2a*, *15* and *CoSUT2* in transgenic apple callus. Asterisks indicate statistical significance (Student's t-test *p<0.05).


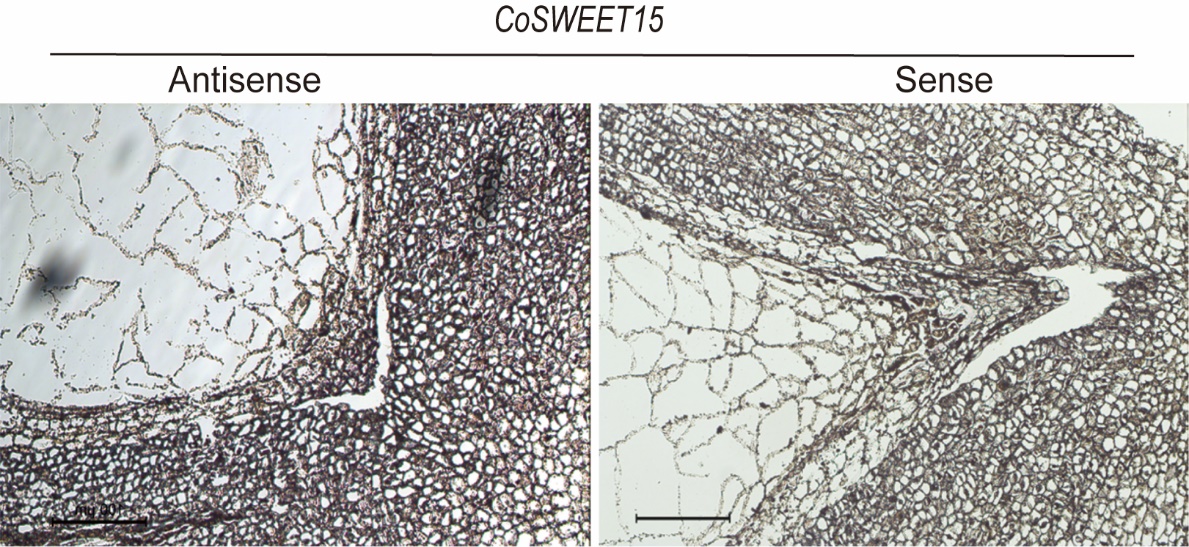


**Figure S10.** In situ hybridizations for *CoSWEET15* in 240 DAP seeds. In situ hybridization of the CoSWEET15 antisense probe (**A**) and sense probe (**B**) showed very low or no background signal. The scale bar is 200 μm.


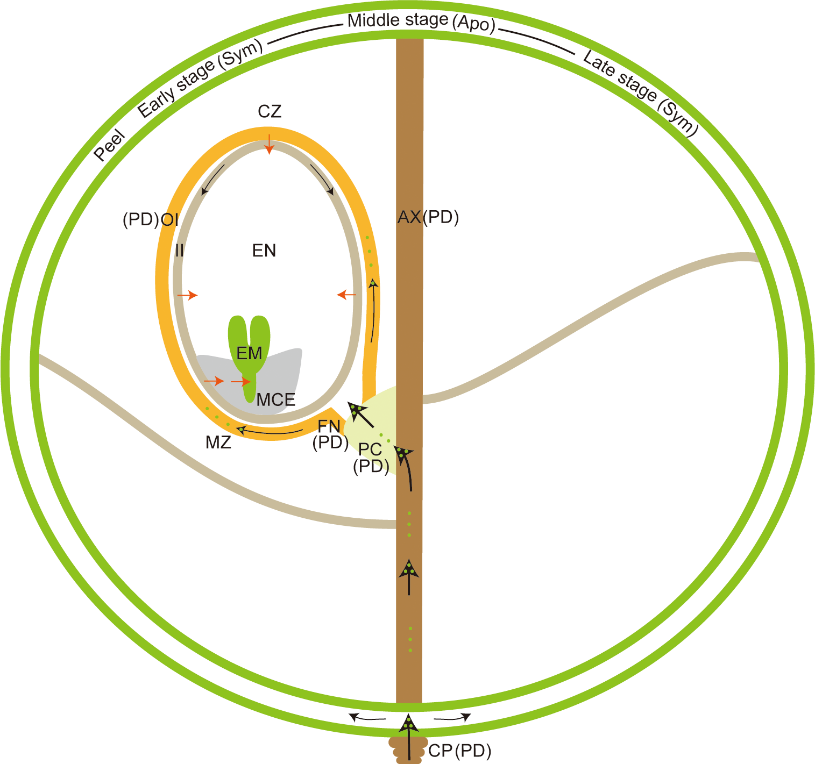


**Figure S11.** Model for sequential symplast-apoplasmic transport steps from carpopodium to embryo in *C. oleifera* fruit. Sucrose, which arrived in the fruit via the carpopodium phloem, can enter the peel and axile through their vascular system. In peel and axile, sucrose was symplasmically unloaded from the phloem and transported to the parenchymal cells through plasmodesmata. Then sucrose arrived in the seed via the placenta phloem which was connected with axile phloem and entered the outer integument via the funicle and seed coat phloem. Similarly, sucrose in these tissues also showed a symplasmic post-phloem unloading. In seed, symplasmic pathway was blocked from the seed coat to endosperm, sucrose can be transported to the endosperm or embryo by the apoplasmic pathway which was initiated by sugar transporters in the chalazal nucellus, transfer cells and micropylar cellularizing endosperm. CP, carpopodium; PD, plasmodesmata; AX, axile; PC, placenta; FN, funicle; OI, outer integument; II, inner integument; MZ, micropylar zone; CZ, chalaza zone; MCE, micropylar cellularizing endosperm; EM, embryo; EN, endosperm; Sym, symplast; Apo, apoplast. The black arrows represent the transport direction of sucrose. The red arrows represent the entry sites of sucrose from maternal to filial tissues. The green points represent sucrose.

**Supplementary Table 1.** The CDS sequences of *CoSWEET1b/2a/15*, *CoSUT2*, *CoCWIN9* and *CoCWIN11*.

| Gene name | Sequence |
| --- | --- |
| *CoSWEET1b* | ATGGGTGGCGTTGCACATTTAGTGTTTGGAGTCTTTGGAAATGCTAATGGGCTGTTGTTGTTTTTGGCACCCACGATAACATTTAAGAGGATTATCATGAACAAATCCACCGAGCGATTCTCAGGCATTCCGTACGTCATGACATTGCTCAACTGCTTGCTTTCTGCTTGGTATGGTCTGCCTTTTGTTTCACCACACAACCTCCTAGTGACAACCATCAATGTCACTGGTGCAGCAATTGAGTCCATCTATGTGTTGATTTTCCTCATTCTTTCACCAAGGAGAGAGAAGGGAAAGATCCTTGGACTTCTTACCGTCGTCCTTGCGATATTCGCCGCGGTTGCCCTCATTTCCTATTTTGCCCTTCATGGCAGAAACCGAACTATTTTTTGCGGTCTAGCTGCCTCCATCTTCTCCATCATAATGTATGCGTCGCCTCTATCGATCATGAGAACGGTGATAAGAACGAAAAGTGTGGAGTACATGCCATTTTTCTTGTCGCTGTTTGTGTTCTTATGCGGTACTTCCTGGTTCGTCTATGGACTGCTTGGAAAGGACCCTTTCATTGCTGTGCCTAACGGTTTTGGATCCGGGTTAGGAACAGTGCAGCTGATCTTGTATGCGATCTACCACAACAACAAGGGTGAAACCAAGAAACCTAGTGTTGATGGATCGGTGGAGATGGAGCTGGGTAAGCCCCAGCAATCACAAAACGGGCATGTCTAG |
| *CoSWEET2a* | ATGGGTGGGTTCTCTAGTCACAATTTGGCTTTCGCATTTGGCCTTTTAGGCAACATCATCTCATTCTTTGTCTTCCTTTCACCCCTGCCAACATTTTACCAAATATTCAAGAAAAAATCAACTGAAGGGTTCCAATCGGTTCCTTACATAGTTGCCTTGTTTAGTGCTATGTTGTTGATGTATTACGCATTTCTCAAGAAGATTGACTCTACACTTATCATCACTATAAACTCATTTGGATGCTTGGTCGAAACTATTTATATTTGTGTTTATCTCTTTTACGCTCCCAAGAAGATTAAGTTCAAAACCATGAAACTACTTGGATTCATGCTTGGCGGGTTTGGCGCGATCCTTATCTTAACTCAATTTCTTATCAAAAAAAGCTCAACCCGCTTTCACATTGTTGGTTGGATTTGCCTATTGTTCTCTGTGAGCGTTTTCGCCGCTCCTCTCTGCATATTGAAACAAGTGATACGAACAAAGAGCGTAGAGTTCATGCCATTTTCCTTATCACTTTCGCTCACGCTTAACGCGGTCATGTGGTTCTTCTATGGCCTTCTAATAAAAGACTTCAACATTGCTATTCCGAATGTCTTGGGGTTTATTTTTGGAATTATTCAAATGGTGCTTTACGCGATATACAAGAACCCAAAGAAAGTTGTTGCGAAAGATCAAAAGTTTTCTTCTGATCAAATACCAAACAAAGTGATAGCCTTGGAGGAAGAGAAGCTATCTGAATTAGTTGAACAAGTGATCGATGTTGTGAAAGATCAAAAGTTTTCTTCTGATCAAATACCAAACAAAGTCATAGCCTTGGAGGAAGAGAAGCTATCTGAATTAGTCGAACAAGTGATTGATGTTGTGAAGCTTAGCTCAATCGCATGTCAAGAAATCGTTCCAGTCATGCCTCATCTGAATGGACACAACATAGCTAGAGAGCTCAATACAATTAAACCAAACCTTGAAGTCACCGTGCCGGTCTAA |
| *CoSWEET15* | ATGGAAATATTTGGTGTTCACCACCCACTGGTGTTTACATTTGGTATCTTAGGTAACATTGTTTCAATCTTTGTGTACCTTGCTCCATTGCCAACATTTATTGAAATATGTAGAAAAAAATCAACTTTAGGGTTTCAATCCCTACCATATGTGGTAGCTCTATTCAGTGCCATGCTTTGGATGTACTATGCATTACTCAAACCAGAAGCAATCCTTCTCATCTCCGTTAACTCATTTGGATGCATCGTAGAGACAATTTACATCATCATTTTCCTTGCTTATGCATCCAAAAAGGCTAAGAACCACACAGCCAAGCTACTTGCTTCTATGAATGTGGGACTCTTCTCTGTGATCCTTCTCATTACACAACTTCTAATTAAAGAGTCATTTCGCGTCTCTGTCATTGGATGGATTTGTGTAGCCATTTCTGTGATCGTTTTTGCAGCACCTCTAAGCATTGTGTTTCATGTTGTCAAAACTCGGAGTGTTGAGTTCATGCCATTTACTTTATCGTTCTTCCTTACATTAAGTGCAATTATGTGGTTCGGTTATGGCCTACTAAAAAAAGACTGGTGTATTGCGCTCCCAAACATATTTGGTTTCTTATTGGGGCTGCTTCAAATGCTTCTATATGGAATATATAGAAATGCTAAGGAAGTTGTGAAGGAGATAAAATTGCCGGAACACATAATCAGCATTGTGATGTTGGGAACCTCTGAAGTACATCCTGTAGATGCTCAAATGCCTAGTGATGATGATAACGATGGGGAAGAAGGTGAGAAGCATGATCAAACAGATCATAACCATGAGAAAAGTGTGGATTTAGCCACGCATGAACGCAGCCAAGCAAATATGCAGCTTGATTCGTCAGTGCTTGTCATGTGTGCAGCTTGA |
| *CoSUT2* | ATGGAGAGTGAGCGACCACCACCATCGGCTTTGGGAGTTCAACAACCGCCGGCGCCGTCGAAATCGTCGCTGCGGAAGACAATAATTGTGGCGTCGATCGCCGCCGGGGTCCAATTCGGGTGGGCCCTACAGCTCTCCCTACTAACACCCTACGTGCAGCTACTGGGCATCCCCCACACTTGGGCCGCCTTCATTTGGCTCTGCGGCCCAATCTCCGGCATGATAGTCCAACCCCTAGTCGGCTACTACAGCGACCGCTGCACTTCCCGATTCGGCCGCCGCCGCCCCTTCATCGCCGCCGGGGCTGGCCTGGTCGCCGTCGCCGTCTTCCTCATCGGTTTCGCCGCCGACCTAGGCCACCTCTCCGGCGACAACCTGGGTCGGACCCCAAAACCCCGAGCCATCGCGGTTTTCGTAGTCGGGTTCTGGATCCTCGACGTCGCCAACAACATGCTCCAAGGCCCTTGCCGGGCCCTCCTGGCGGATCTTTCCGGCGACAACCAACGGAAAATGCGAACCGCCAACTCTTTATATTCCTTCTTCATGGCGGTCGGGAACGTTCTCGGGTTCGCCGCCGGGTCATATACCCATCTCCACAAAGCCTTCCCATTCTCATCCACACGTGCATGTGATGTGTACTGTGCGAACCTCAAGAGCTGTTTCTTTATCTCAATCGCTCTCTTAATCACTCTAACCACCATAGCTCTAACAACAGTGCGTGAAAAACCCATCTCCAAACCGGAACCGGGACATACCGGTGACTTGGACGGGGTCGGCAAGGATGGGGAAAAGGCTAAGCCGGTGCCGTTCTTCGGAGAAATATTCGCTGCGCTGAAGGACTTGCCTAGGCCCATGTGGATCTTGCTTCTGGTGACGTGTCTTAATTGGATTGCTTGGTTCCCTTTCTTGTTGTTCGACACGGATTGGATGGGCAGGGAGGTGTACGGAGGGAAGGTTGGGGAAGGACAGGCTTACGATCACGGCGTCCGCGCGGGTGCATTGGGGCTGATGCTGAACTCGGTGGTGTTGGGGTTCATGTCACTCGCGGTGGAGCACTTGGCTCGTGGGCTCGGCGGGGTCAAGCGCTTGTGGGGTGTGGTCAACTTTTTGCTGGCGATTTGTCTGGCCATGACGGTGGTGATTACCAAAAAAGCAGAGTCCGCACGCCATTTCGCCGCCGCACACAACGGAGGAGCCACCCCTTCGCATCCTGATACCGGCGTCAAGGTCGGCGCGTTGACACTCTTCTCCGTTCTCGGAATCCCTCTAGCGGTGACTTACAGCATTCCGTTTGCTCTAGCATCCATTTTCTCAAACAGTGCTGGTGCAGGGCAAGGTCTCTCTCTGGGAGTTCTGAATCTTGCAATAGTTATCCCACAGATGGTGGTGTCAGTAGCAAGTGGACCGTGGGATGCCTTATTCGGGGGTGGCAACTTACCTGCGTTTGTTGTAGGTGCGGTTGCAGCGGCAGCGAGCGGGGTGTTTGCAGTTACCATGCTTCCATCTCCGCCACCTGATACTGCATCCACCAAAGTTCCCATGGCTATTGCTGCATTCCATTGA |
| *CoCWIN9* | ATGTACTACAATGGAACATACCATTTCTTTTACCAGTACAATCCCAAAGGTGCAGTTTGGGGCAACATTGTGTGGGCCCATTCGGTATCGACGGACCTCATCAATTGGAAGCCACTTCCTCCAGCAATCTACCCTTCAAAGCCGTTCGATCAATACGGCACTTGGTCTGGGTCGGCCACAATTCTCCCAAGCAACAAGCCCATCATACTCTACACTGGGATAATTGATAAAAACAACACACAAGTCCAAAACTATGCAATACCCGCTAACTATTCGGATCCATACCTCCAAAAATGGATAAAACCCAACAACAACCCGTTAGTGTTCCCGGTCGGTGTGAATAAAACCGCTTTTCGTGGCCCGACAACTGCATGGTTCGGCAAAGATAGGCATTGGAGGATTTTGGTGGGAGGAAGGAGAAAACATAGAGGGATGGCCCATTTGTATCGGAGTAAAGACTTCATGAAGTGGACTAAGGCCCAATACCCATTGCATTCTTCGGCCATGACGGGTATGTGGGAATGCCCCGACTTTTACTCGGTATCTTTGGATTCTGAAAATGGGTTAGAGGTTTCTGTGATTGAGGGAAATATTAAGCATGTTTTAAAGGTGAGTCTGGATGAAACAAGATATGAGTATTATACAATTGGAAAATATTTCTGGAAGATTGATAAATATGTACCAAATAATACATTCATTGATAGTTGGGCCGGGCTGAGATATGATTATGGAAATTTGTATGCATCAAAGACATTTTTCGATGCTGGAAAGAATCGGAGAATCTTGTGGGGGTGGGCTAATGAGTCAGATACTTCTATTGAAGATGTTAAAAAAGGTTGGGCTGGAATTCAGACAATTCCAAGAAAGGTCTGGCTTGATAAAAGTGAAAAACAGTTAGTACAATGGCCGGTTGAAGAATTAGAAACTCTAAGGAGAAGAAAGGTTCACCTAAGTAATCAAAAGCTCAATTTGGGAGACCATGTTGAAATCAAAGAAATTACAGCTGCACAGGCTGATGTTGAAGTGACATTCTCATTCCCAAGTTTGGACAAAGCTGAGGCATTTGACCCTAGTTGGGCCAAAGACAATCATGTAGTTCTCATGTGCTCTGATGCTACAAGTTCCTCTTTGAAGAAAAAGCTGTACAAGCCATTATTTGCAGGTTTTGTGAATGTGGATTTAGCACAAAACATACTTTCCCTTCGAAGTTTGATTGATAATTCTGTTGTGGAAAGTTTTGGTGCTGGAGGAAAAACATGCATAACATCTAGGGTTTATCCAACTCTAGCAGTATTCAAGGATGCTCATTTATATGTATTCAACAATGGTACTGAAACGGTCACAGTTGATAATCTGGATGCCTGGAGCATGAACAATCCTTTAATGAATTAA |
| *CoCWIN11* | ATGGAGTACCAGTCTGTTTCTGCTATTGAACTCAATCACCTCCACAGAACTGGCTTCCACTTTCAACCCAAAAAGCACTGGATCAATGGACCAATGTACTACAATGGAATATACCATTTCTTTTACCAGTACAACCCCAAAGGTGCAGTTTGGGGCAACATTGTGTGGGCCCATTCGGTATTGACGGACCTCATCAATTGGAAGCCACTTCCTCCAGCAATCTACCCTTCAAAGCCGTTCGATCAATACGGTACTTGGTCTGGGTCAGCCACAATTCTCCCAGGCAACAAGCCCATCATACTCTACACTGGGATAATTGATAAAAATAACACACAAGTCCAAAACTATGCAATACCCACTAACTATTCGGATCCATACCTCCAAAAATGGATAAAACCCAACAACAACCCGTTAGTGTCCCCGGTCGGTGTGAATAAAACCGCTTTCCGTGACCCGACAACTGCATGGTTCGGCAAAGATGGGCATTGGAGGATGTTGGTGGGAGGAAGGAGAAAACATAGAGGGATGGCCCATTTGTATCGGAGTAGAGACTTCATGAAGTGGACTAAGGCCCAACACCCATTGCATTCTTCGGCCATGACGGGTATGTGGGAATGCCCCGACTTTTACCCGGTATCTTTGGATTCTGAAAATGGGTTGGAGGTTTCTGTGATTGAGGGAAATATTAAGCATGTTTTAAAGGTGAGTCTGGATGAAACAAGATATGAGTATTATACAATTGGAAAATATTTCTCGAAGATTGATAAATATGTACCAGATAATACATCCGTTGATAGTTGGGCCGGGTTGAGATATGATTATGGAAATTTTTATGCATCAAAGACATTTTTCGATGCTGGAAAGAATCGGAGAATCTTGTGGGGGTGGGCTAATGAGTCAGATACTTCTATTGAAGATGTTAAAAAAGGTTGGGCTGGAATTCAGACAATTCCAAGAAAGGTCTGGCTTGATAAAAGTGAAAAACAGTTAGTACAATGGCCGGTTGAAGAATTAGAAACTCTAAGGAGGAGAAAGGTTCACCTAAGTAATCAAAAGCTCAATTTGGCAGACCATGCTGATGTTGAAGTGACATTCTCATTCCCAAGTATGGACAAAGCTGAGGCATTTGACCCTAGTTGGGTTAACATGGATGCCCAAGAACTTTGTAAACACAAGGGTTCAACTGTTCAAGGTGGGGTTGGACCATTTGGGCTTCTGACATTGGCTTCACAACACCTTGAAGAATACACTCCTGTTTTCTTTAGGGTTTTTAAGGCCAAAGACAATCATGTAGTTCTCATGTGCTCTGATGCTACAAGTTCCTCTTTGAAGAAAAAGCTGTACAAGCCATCATTTGCAGGTTTTGTGAATGTGGATTTAGCACAAAACAAACTTTCCCTTCGAAGTTTGATTGATAATTCTGTTGTGGAAAGTTTTGGTGTTGGAGGAAAAACATGCATAACATCTAGGGTTTATCCAACTCTAGCAGTATGCAAGGATGCTCATTTATATATATTCAACAATGGTACTGAAACGGTCACAGTTGAGAATCTGGATGCCTGGAGCATGAACTATCCTTTAATGAACTAA |

**Supplementary Table 2.** List of primers used for the study.

| Primer name | Primer Sequence | Primer Use |
| --- | --- | --- |
| CoSWEET1b-F | ATGGGTGGCGTTGCACATTTAG | Gene clone |
| CoSWEET1b-R | CTAGACATGCCCGTTTTGTGATTGC | Gene clone |
| CoSWEET2a-F | ATGGGTGGGTTCTCTAGTCACAAT | Gene clone |
| CoSWEET2a-R | TTAGACCGGCACGGTGACT | Gene clone |
| CoSWEET15-F | GAGAGAGTTTGATTGAAGAGATGG | Gene clone |
| CoSWEET15-R | ATGTAATATCATGTAAATGGAGCCG | Gene clone |
| CoSUT2-F | ATGGAGAGTGAGCGACCACCACC | Gene clone |
| CoSUT2-R | CAATGGAATGCAGCAATAGCCATCG | Gene clone |
| CoSWEET1b-qF | AGGAGAGAGAAGGGAAAGATCC | qRT-PCR |
| CoSWEET1b-qR | AATAGTTCGGTTTCTGCCATGAAG | qRT-PCR |
| CoSWEET2a-qF | GTTGTTGCGAAAGATCAAAAGTT | qRT-PCR |
| CoSWEET2a-qR | TTCTTGACATGCGATTGAGCTAA | qRT-PCR |
| CoSWEET15-qF | ATATTTGGTTTCTTATTGGGGCT | qRT-PCR |
| CoSWEET15-qR | TGGCTAAATCCACACTTTTCTCA | qRT-PCR |
| CoSUT2-qF | TGGACCGTGGGATGCCTTAT | qRT-PCR |
| CoSUT2-qR | TCAATGGAATGCAGCAATAGCC | qRT-PCR |
| CoGAPDH-qF | GGTGCCAAGAAGGTGGTAATA | qRT-PCR |
| CoGAPDH-qR | GTTGTGCAGCTTGCATTAGAG | qRT-PCR |
| CoActin-qF | AAACTACGGTTGCGGATAGAG | qRT-PCR |
| CoActin-qR | CTCCGGTGCATCCTTCATAAT | qRT-PCR |
| CoSWEET1b- pCAMBIA1300-F | GGGGCCCGGGGTCGACATGGGTGGCGTTGCAC | Subcellular localization and Transformation of apple callus |
| CoSWEET1b- pCAMBIA1300-R | CCATGGTACCGGATCCGACATGCCCGTTTTGT | Subcellular localization and Transformation of apple callus |
| CoSWEET2a- pCAMBIA1300-F | GGGGCCCGGGGTCGACATGGGTGGGTTCTCTAG | Subcellular localization and Transformation of apple callus |
| CoSWEET2a- pCAMBIA1300-R | CCATGGTACCGGATCCGACCGGCACGGTGAC | Subcellular localization and Transformation of apple callus |
| CoSWEET15- pCAMBIA1300-F | GGGGCCCGGGGTCGACATGGAAATATTTGGTG | Subcellular localization and Transformation of apple callus |
| CoSWEET15- pCAMBIA1300-R | TGCTCACCATGGTACCAGCTGCACACATGACA | Subcellular localization and Transformation of apple callus |
| CoSUT2- pCAMBIA1300-F | GGGGCCCGGGGTCGACATGGAGAGTGAGCGAC | Subcellular localization and Transformation of apple callus |
| CoSUT2- pCAMBIA1300-R | CCATGGTACCGGATCCATGGAATGCAGCAAT | Subcellular localization and Transformation of apple callus |
| CoSWEET1b- P2YN-F | CGCCACTAGTGGATCCATGGGTGGCGTTGCAC | BiFC |
| CoSWEET1b- P2YN-R | TCCCGGGAGCGGTACCGACATGCCCGTTTTGT | BiFC |
| CoSWEET1b- P2YC-F | CGCCACTAGTGGATCCATGGGTGGCGTTGCAC | BiFC |
| CoSWEET1b- P2YC-R | TCCCGGGAGCGGTACCGACATGCCCGTTTTGT | BiFC |
| CoSWEET2a- P2YN-F | CGCCACTAGTGGATCCATGGGTGGGTTCTCTAG | BiFC |
| CoSWEET2a-P2YN -R | TCCCGGGAGCGGTACCGACCGGCACGGTGAC | BiFC |
| CoSWEET2a- P2YC-F | CGCCACTAGTGGATCCATGGGTGGGTTCTCTAG | BiFC |
| CoSWEET2a- P2YC-R | TCCCGGGAGCGGTACCGACCGGCACGGTGAC | BiFC |
| CoSWEET15- P2YN-F | CGCCACTAGTGGATCCAGAGAGAGTTTGATT | BiFC |
| CoSWEET15-P2YN -R | TCCCGGGAGCGGTACCATGTAATATCATGTAA | BiFC |
| CoSWEET15- P2YC-F | CGCCACTAGTGGATCCAGAGAGAGTTTGATT | BiFC |
| CoSWEET15-P2YC-R | TCCCGGGAGCGGTACCCGATGTAATATCATG | BiFC |
| CoSUT2-P2YN-F | CGCCACTAGTGGATCCATGGAGAGTGAGCGA | BiFC |
| CoSUT2-P2YN -R | TCCCGGGAGCGGTACCTCAATGGAATGCAGC | BiFC |
| CoSUT2-P2YC-F | CGCCACTAGTGGATCCATGGAGAGTGAGCGA | BiFC |
| CoSUT2-P2YC-R | TCCCGGGAGCGGTACCCGTCAATGGAATGCAGC | BiFC |
| CoSWEET1b- pDR196-F | CGAGCTCAACTTCGAAATGGGTGGCGTTGCAC | Complementation of yeast |
| CoSWEET1b- pDR196-R | TTCCCTCGAGGTCGACCTAGACATGCCCGTTT | Complementation of yeast |
| CoSWEET2a- pDR196-F | CGAGCTCAACTTCGAAATGGGTGGGTTCTCTAG | Complementation of yeast |
| CoSWEET2a- pDR196-R | TTCCCTCGAGGTCGACTTAGACCGGCACGGTGAC | Complementation of yeast |
| CoSWEET15- pCAMBIA1300-F | CCGGAATTCATGGAAATATTTGGTGTTCACC | Complementation of yeast |
| CoSWEET15- pDR196-R | CCGCTCGAGTCAAGCTGCACACATGACAAG | Complementation of yeast |
| CoSUT2-pDR196-F | CGAGCTCAACTTCGAAATGGAGAGTGAGCGAC | Complementation of yeast |
| CoSUT2-pDR196-R | TTCCCTCGAGGTCGACTCAATGGAATGCAGCA | Complementation of yeast |
| AtSUC2-pDR196-F | CGAGCTCAACTTCGAAATGGTCAGCCATCCA | Complementation of yeast |
| AtSUC2-pDR196-R | TTCCCTCGAGGTCGACTCAATGAAATCCCAT | Complementation of yeast |
| SiSWEET7a-pDR196-F | CGAGCTCAACTTCGAAATGACTTTTAATAG | Complementation of yeast |
| SiSWEET7a-pDR196-R | TTCCCTCGAGGTCGACTGAAAAACAGAAGGCCCAAT | Complementation of yeast |
| CoCWIN9-qF | CCCTAGTTGGGCCAAAGACAAT | qRT-PCR |
| CoCWIN9-qR | ACTGTGACCGTTTCAGTACCA | qRT-PCR |
| CoCWIN11-qF | AATGTTGCCCCAAACTGC | qRT-PCR |
| CoCWIN11-qR | CCTCCACAGAACTGGCTTCC | qRT-PCR |
| CoCIN1-qF | GAGCCCGGGATCTTCTTGAAA | qRT-PCR |
| CoCIN1-qR | ACGGCCAATAGCTGACTCAC | qRT-PCR |
| CoCIN8-qF | TTGGCCAACCCTTTTATGGCA | qRT-PCR |
| CoCIN8-qR | CGGGCTTGCTTGCCAACG | qRT-PCR |
| CoVIN5-qF | GGGTATTGGGTTACGGGTGG | qRT-PCR |
| CoVIN5-qR | TGTTGCCTCCAATGCTCTGA | qRT-PCR |
| CoINH5-qF | CCCTCAATTTTGGGGACAC | qRT-PCR |
| CoINH5-qR | GCGAGGGTCTGACCTTAGAG | qRT-PCR |
| CoSPS1-qF | TGATGCTATGGAGAACTGGGC | qRT-PCR |
| CoSPS1-qR | TCATGGATGTCATTTTCCAGGTT | qRT-PCR |
| CoSPS4-qF | TGTTCGTTGGGGAAAGAGGG | qRT-PCR |
| CoSPS4-qR | GGGCAATGAATGCGAAGACC | qRT-PCR |
| CoSUS1-qF | GGTACCGCAATGGAGAGCTG | qRT-PCR |
| CoSUS1-qR | TTGCTCGATTCATCATCGTCA | qRT-PCR |
| CoSSS2-qF | TCCATTGCCACGATTGGTCT | qRT-PCR |
| CoSSS2-qR | TGAGGAGCAATTGCAGTGTTT | qRT-PCR |
| CoGBSS4-qF | GCACTGGAAACCCTACCTCC | qRT-PCR |
| CoGBSS4-qR | GATGGCAGCTCATCAGACCA | qRT-PCR |

**Supplementary Table 3.** The specific gene fragment in *CoSWEET1b/2a/15*, *CoSUT2*, *CoCWIN9* and *CoCWIN11*.

| Primer name | Specific Fragment |
| --- | --- |
| *CoSWEET1b* | AGGAGAGAGAAGGGAAAGATCCTTGGACTTCTTACCGTTGTCCTTGCGATATTCGCGGCGGTTGCCCTCATTTCCTATTTTGCCCTTCATGGCAGAAACCGAACTATTTTTTGCGGTCTAGCTGCCTCGATCTTCTCCATCATCATGTACGCGTCGCCTCTATCAATCATGAGAACGGTGATAAGAACGAAAAGTGTGGA |
| *CoSWEET2a* | GTTGTTGCGAAAGATCAAAAGTTTTCTTCTGATCAAATACCAAACAAAGTGATAGCCTTGGAGGAAGAGAAGCTATCTGAATTAGTTGAACAAGTGATCGATGTTGTGAAAGATCAAAAGTTTTCTTCTGATCAAATACCAAACAAAGTCATAGCCTTGGAGGAAGAGAAGCTATCTGAATTAGTCGAACAAGTGATTGATGTTGTGAAGCTTAGCTCAATCGCATGTCAAGAAATCATTCCAGTCATGCCTCATCTGAATGGACACAACATAGCTAAAGAGCTCAATACAATTAAACCCAACCTTGAAGTCACCGTGCCGGTCTAA |
| *CoSWEET15* | TCACCACCCACTGGTGTTTACATTTGGTATCTTAGGTAACATTGTTTCAATCTTTGTGTACCTTGCTCCATTGCCAACATTTATTGAAATATGTAGAAAAAAATCAACTTTAGGGTTTCAATCCCTACCATATGTGGTAGCTCTATTCAGTGCCATGCTTTGGATGT |
| *CoSUT2* | CTGATGCTGAACTCGGTGGTGTTGGGGTTCATGTCACTCGCGGTGGAGCACTTGGCTCGTGGGCTCGGCGGGGTCAAGCGCTTGTGGGGTGTGGTCAACTTTTTGCTGGCGATTTGTCTGGCCATGACGGTGGTGATTACCAAAAAAGCAGAGTCCGCACGCCATTTCGCCGCCGCACACAACGGAGGAGCCACCCCTTCGCATCCTGATACCGGCGTCAAGGTCGGCGCGTTGACACTCTTCTCCGTTCTCGGAATCC |
| *CoCWIN9* | TCGGGAGTTCAAGCGATTCCAAGGAAAATTTGGCTCGATAAATCTGGGAAACAATTGATGCAATGGCCGATTGCAGAGATAGAAGAGCTACGAAAGAACCAAGTTGACATGCCTAGCATGGTGCTGAAGGGAGGGTCGGTGGTTGAAGTTTCTGGTGTCACAGGCACACAGGCAGATGTA |
| *CoCWIN11* | TGACGGGTATGTGGGAATGCCCGGATTTCTACCCTGTATCTTTGGATTGTGAAAACGGGTTGGAAACATCAGTGATGACAGGAAATATTAAGCATGTCTTAAAAGTGAGCCTTGATGAAACTAGATATGAATACTATACAGTTGGAACATATTTTCCCAAGAAGGATAGTTATATACCAGATAATACTTCTGTTGATGGTCGAGCC |
